# Effects of Dual-Eligible Integrated Care Plans on Medicaid Enrollment and Retention: Evidence From the Implementation of Medicare-Medicaid Plans

**DOI:** 10.1177/10775587251372267

**Published:** 2025-10-02

**Authors:** Eric T. Roberts, Eliza Macneal, Kenton J. Johnston, José F. Figueroa

**Affiliations:** 1 University of Pennsylvania, Philadelphia, USA; 2 Washington University in St. Louis, MO, USA; 3 Harvard T.H. Chan School of Public Health, Boston, MA, USA; 4 Brigham and Women’s Hospital, Boston, MA, USA

**Keywords:** dual-eligible beneficiaries, integrated care programs, Medicaid take-up

## Abstract

Medicare and Medicaid are separate programs that together cover 13 million low-income older adults and people with disabilities, known as dual-eligible individuals. Concern about a lack of coordination across Medicare and Medicaid has prompted the development of Integrated Care Programs (ICPs). Although the primary goal of ICPs is to coordinate financing and care across Medicare and Medicaid, ICPs may also influence whether low-income individuals obtain or keep Medicaid. We evaluated whether the rollout of Medicare-Medicaid Plans (MMPs)—one of the largest ICPs—was associated with changes in Medicaid take-up and retention among Medicare beneficiaries residing in high-poverty zip codes. Using a stacked difference-in-differences design and variation in MMP rollouts across nine states, we found no evidence that MMPs increased monthly or continuous Medicaid enrollment in this population. These findings highlight the need for focused policies to address Medicaid enrollment gaps among low-income Medicare beneficiaries, which could complement broader integration efforts.

## Introduction

Approximately 13 million low-income older adults and people with a disability or qualifying medical condition are dually eligible for and enrolled in Medicare and Medicaid. Medicare and Medicaid each cover different services for dual-eligible beneficiaries, and for most, the programs are not coordinated (Grabowski, 2007; Peña et al., 2024). Medicare, a federal program, is the primary payer for prescription drugs and inpatient, outpatient, and post-acute care. Medicaid, which is managed by states with joint federal-state funding, covers Medicare out-of-pocket costs and, for 9 million individuals with full Medicaid, it also provides coverage for long-term care and additional behavioral health services (Medicare Payment Advisory Commission & Medicaid and CHIP Payment and Access Commission, 2024).

The bifurcated nature of Medicare and Medicaid poses numerous administrative and caregiving challenges (Bella et al., 2024). For example, beneficiaries must navigate different eligibility pathways for Medicare and Medicaid (Feng et al., 2019a; Johnston et al., 2024). Eligibility for Medicare is based on age, presence of a qualifying disability, or end-stage renal disease. Once individuals enroll in Medicare, most remain continuously covered while alive. Conversely, Medicaid eligibility is based on income and assets, and most beneficiaries must complete an annual eligibility redetermination (Burns et al., 2024). Administrative complexity and the lack of a coordinated process for managing Medicaid renewals may contribute to low enrollment and result in individuals cycling on and off Medicaid, termed “churn” (Feng et al., 2019b; Lakhmani et al., 2022; Tjhia et al., 2024). Prior studies estimated that as many as one-half of Medicare beneficiaries who qualified for Medicaid did not enroll and that 7% to 8% of dual-eligible beneficiaries disenrolled from Medicaid annually (Ma et al., 2024; Pezzin & Kasper, 2002; Riley et al., 2014). Of those who disenrolled, approximately one-half regained Medicaid within 12 months (Ma et al., 2024). Furthermore, beneficiaries and caregivers often have trouble navigating services covered by separate programs (e.g., Medicare-funded post-acute care and Medicaid-funded long-term care), and few administrative or financial levers exist to support care coordination (Grabowski, 2007).

To address these challenges, policymakers are prioritizing efforts to expand integrated care plans (ICPs) (Bella et al., 2024; Medicare Payment Advisory Commission, 2019; Velasquez et al., 2023). ICPs are models in which one entity—typically a managed care plan—manages Medicare and Medicaid benefits and spending for dual-eligible beneficiaries. To date, policymakers have had experience with three major categories of ICPs: (1) Medicare-Medicaid Plans (tested under the Financial Alignment Demonstration of the Centers for Medicare and Medicaid Services), (2) Fully Integrated Managed Dual Eligible Special Needs Plans (a category of Medicare Advantage Dual Eligible Special Needs Plans), and (3) Programs of All-Inclusive Care for the Elderly (for older adults needing nursing home-level care) (Medicaid and CHIP Payment and Access Commission, 2022a; Medicaid and CHIP Payment and Access Commission, 2022b; Roberts et al., 2024). ICPs primarily enroll dual-eligible beneficiaries with full Medicaid, reflecting this population’s need for coordinated Medicare and Medicaid services (Bella et al., 2024). Although ICPs have different financial and organizational characteristics, common goals are to coordinate services within one entity that bears risk for Medicare and Medicaid spending, reduce costly hospital and nursing home use, and expand access to community-based services and supports. ICPs may also simplify how beneficiaries access Medicare and Medicaid benefits by providing a single point of contact to coordinate coverage (Greene, 2017; Medicaid and CHIP Payment and Access Commission, 2021; Mellor et al., 2024).

Conceptually, there are several ways ICPs might affect low-income Medicare beneficiaries’ enrollment in and retention of Medicaid. First, ICPs have incentives to reduce Medicaid churn because the plans can only enroll dual-eligible beneficiaries and are paid on a capitated basis (i.e., a risk-adjusted amount per enrollee-month) (Medicare Payment Advisory Commission, 2018). Reducing churn helps ICPs attain a predictable revenue stream and maintain a sufficient enrollment base to pool risks. It may also mitigate adverse selection by reducing the likelihood that Medicaid enrollment is concentrated during costly care episodes—a pattern observed in prior research, which found that Medicare beneficiaries are more likely to enroll in Medicaid during periods of expensive hospital or nursing facility care (Garrow et al., 2023; Keohane et al., 2017; Spillman & Waidmann, 2014). Second, helping individuals retain Medicaid can enable ICPs to provide ongoing care management and supportive services, such as Medicaid home- and community-based services, for people with long-term care needs. Providing these services continuously could avert the need for costly downstream care associated with unmet need, such as hospitalizations (Allen et al., 2014; Wolff et al., 2019), and MMPs could capture the resulting savings if they expect to retain enrollees over time. Third, by coordinating coverage within a single plan, MMPs may be able to reduce the administrative burden of Medicaid renewals. For example, MMPs can notify enrollees about upcoming Medicaid renewals, assist with renewal paperwork, and serve as a central point of contact to address coverage questions (Lakhmani et al., 2022).

Evaluating whether ICPs increase Medicaid enrollment and reduce churn is important for several reasons. First, it can illuminate whether ICPs are effective in helping individuals navigate administrative hurdles to obtaining or keeping Medicaid. This is especially relevant for individuals from socially disadvantaged populations, who often face challenges navigating health insurance (Kyle & Frakt, 2021; Office of Management and Budget, 2021). Second, it can shed light on whether ICPs may be effective in helping enrollees maintain Medicaid coverage that provides access to long-term services and supports. Third, it can help policymakers better anticipate the costs of expanding ICPs, as has been envisioned in recent legislative proposals (Sen Cassidy, 2024). For example, Medicaid costs could rise if there is an increase in Medicaid-covered months, and capitation payments to ICPs, that are not offset by efficiencies in how ICPs manage care. Cost impacts also depend on whether marginal enrollees—those taking up Medicaid due to ICP expansions—have different costs from existing Medicaid enrollees. However, empirical evidence about the impacts of ICPs on these margins remains limited.

## New Contribution

This study examines whether the implementation of MMPs was linked to increases in Medicaid enrollment among Medicare beneficiaries, including increases in continuous enrollment (an indicator of reduced Medicaid churn). The MMPs are advantageous to study as they were among the largest ICPs tested in the last decade and their implementation varied across states. We leverage this variation in a quasi-experimental difference-in-differences design and use administrative data to assess monthly Medicaid enrollment. To focus on a population that was most likely to include Medicaid-eligible individuals, we analyzed Medicare beneficiaries residing in zip codes where a high proportion of older adults had incomes below the federal poverty level.

## Policy Context

Medicare-Medicaid Plans (MMPs) are financially integrated managed care plans for dual-eligible beneficiaries. The plans were implemented in 10 states as part of the Medicare-Medicaid Financial Alignment Initiative (FAI), a program of the Innovation Center at CMS and the Medicare-Medicaid Coordination Office that tested integrated models for dual-eligible beneficiaries (Medicare Payment Advisory Commission, 2018). In addition to MMPs, the FAI tested managed fee-for-service models in Colorado and Washington, which we did not study. States tested MMPs for different periods. For example, Virginia’s MMP model operated from April 2014 to December 2017 and California’s ran from April 2014 to December 2022. Other states continued their programs through 2025, when all remaining MMPs are scheduled to end.

MMPs received pooled capitation payments covering all Medicare- and Medicaid-funded services for dual-eligible beneficiaries (Centers for Medicare and Medicaid Services, 2019). In all states, enrollment was limited to dual-eligible beneficiaries with full Medicaid who lived in counties that were part of states’ MMP demonstration areas. Some states further limited enrollment to specific beneficiary subpopulations based on characteristics such as age (e.g., Massachusetts and South Carolina) or specific needs, such as long-term care or presence of an intellectual or developmental disability (e.g., New York).

Enrollment in MMPs was voluntary. States and plans used a combination of active enrollment (e.g., marketing and recruitment of beneficiaries) and passive enrollment, wherein individuals residing in MMP demonstration areas were automatically enrolled in the plans but could opt out at any point (Medicare Payment Advisory Commission, 2016). Due to varying opt-out rates, participation in the MMPs differed across states, from 62% in Ohio to 5% in New York (Grabowski et al., 2017).

Table 1 summarizes the geographic scope, implementation dates, eligible populations, and enrollment in MMPs by state. MMPs were offered in seven and six large metropolitan counties in California and Texas, respectively. In Illinois, Michigan, and Ohio, MMPs were offered in approximately 20% to 30% of counties, including most major cities. MMPs were offered in 77% of counties in Virginia, and all or almost all counties in Massachusetts, Rhode Island, and South Carolina. In all but three states, MMPs served all dual-eligible beneficiaries regardless of age or health condition. In Massachusetts, enrollment was limited to beneficiaries under age 65; in South Carolina, enrollment was limited to those ages 65 and older (Barnette, 2021). In New York, which had the fewest enrollees, two MMPs served dual-eligible individuals needing nursing facility-level care and people with intellectual and developmental disabilities (Centers for Medicare and Medicaid Services, n.d.-a).

**Table 1.** Summary of Medicare-Medicaid Plans (MMPs) and Analytical Comparison Regions.

State	Demonstration areas	Target population(s)	Implementation date	Number (% of eligible) enrollees in third year	Comparison region(s) analyzed^a^	Neighbor state(s) used as comparisons
California	San Mateo, Santa Clara, Los Angeles, Orange County, San Bernardino, Riverside, and San Diego counties	All dual eligibles	April 1, 2014	112,201 (23.0%)	In-state, neighbor state	AZ, NV, OR
Illinois	21 counties in the central and northeast regions	All dual eligibles	March 1, 2014	46,294 (30.2%)	In-state, neighbor state	IA, KY
Massachusetts	12 of 14 counties	Dual eligibles aged <65 years	October 1, 2013	12,286 (12.0%)	Neighbor state	CT, NH, NY^b^, VT
Michigan	25 counties in the south and upper peninsula regions	All dual eligibles	March 1, 2015	38,259 (35.0%)	In-state	
Ohio	29 counties in the northeast, northwest, central, and southwest regions	All dual eligibles	May 1, 2014	69,331 (68.8%)	In-state, neighbor state	KY, PA, WV
Rhode Island	Whole state	All dual eligibles	July 1, 2016	15,555 (40.0%)	Neighbor state	CT
South Carolina	44 of 46 counties	Dual eligibles aged ≥65 years	February 1, 2015	11,511 (55.5%)	Neighbor state	GA, NC
Texas	Bexar, Dallas, El Paso, Harris, Hidalgo, and Tarrant counties	All dual eligibles	March 1, 2015	43,660 (28.1%)	In-state, neighbor state	KS, MO, MS, OK
Virginia	102 of 133 counties	All dual eligibles	April 1, 2014	28,700 (40.1%)	In-state, neighbor state	NC, TN
New York (FIDA)	Bronx, Kings, Nassau, New York, Queens, Richmond, Suffolk, and Westchester counties	Dual eligibles requiring nursing facility-level care	January 1, 2015	4,158 (3.2%)	Not analyzed due to low enrollment	
New York (FIDA-IDD)	Bronx, Kings, Nassau, New York, Queens, Richmond, Rockland, Suffolk, and Westchester counties	Dual eligibles with intellectual or developmental disabilities	April 1, 2016	1,128 (5.3%)	Not analyzed due to low enrollment	

*Source.* CMS Financial Alignment Initiative reports.

*Note.* ^a^In-state comparison regions are non-MMP counties within MMP states. Neighbor state comparison regions are non-MMP states that neighbor MMP states, matched on ACA Medicaid expansion status (see methods). FIDA, Fully Integrated Duals Advantage; IDD, Intellectual and Developmental Disabilities.

b New York had two MMP demonstrations, although both enrolled few individuals (see last two rows of table). We included New York as a comparison state for Massachusetts because the modest scale of New York’s program makes it unlikely to meaningfully attenuate regression estimates.

Our analysis focused on MMPs in nine states: California, Illinois, Massachusetts, Michigan, Ohio, Rhode Island, South Carolina, Texas, and Virginia. We did not include New York in the intervention group due to the small scale of its MMPs.

## Method

### Data and Sample

Our primary data source was the Medicare Master Beneficiary Summary File (MBSF), which we analyzed for a 20% random sample of Medicare beneficiaries from 2010 to 2019. We used the MBSF to measure beneficiary demographics (age, race and ethnicity, and sex), original reason for Medicare entitlement (age, disability, or end-stage renal disease), geography (state, county, and zip code), and monthly Medicaid enrollment. We used FAI reports on the CMS website to identify the dates of MMP rollouts and the counties where these plans operated.

As the MBSF only reports Medicaid *enrollment* but not eligibility, we used residence in a high-poverty zip code, defined as the highest quintile of poverty among people aged 65 and older (based on 5-year averages from the 2019 American Community Survey), to identify areas where low-income Medicare beneficiaries were most likely to live. This approach aligns with other recent research (Bundorf et al., 2024). To isolate changes in Medicaid take-up unrelated to Medicare coverage (e.g., Medicaid eligibility changes when people age into Medicare), we further limited the sample to people with at least 2 years Medicare coverage prior to the analysis year. We included person-month data on Medicaid enrollment in the 36 months before and 36 months after MMP implementation and contemporaneous periods in comparison groups (see Supplemental Appendix Figure 1 for a sample inclusion flow diagram). We confirmed that MMP enrollment among dual-eligible beneficiaries tracked state-specific rollout dates for these plans (Supplemental Appendix Figure 2).

### Study Design and Treatment and Comparison Groups

We used a difference-in-differences (DID) design to assess whether the introduction of MMPs was associated with increased Medicaid enrollment and retention. We first estimated separate treatment effects for each MMP state and then aggregated treatment effects across states. All analyses followed an intention-to-treat approach, comparing beneficiaries who met our inclusion criteria and resided in MMP counties (treatment group) to those in non-MMP counties (comparison group).

We conducted complementary analyses using two sets of comparison groups. The first comparison group included beneficiaries living in states without MMPs that neighbored states with MMPs *(neighbor state comparisons)*. To control for potential spillovers of Affordable Care Act (ACA) Medicaid expansions, which targeted working-age adults *without* Medicare, on Medicaid enrollment among individuals *with* Medicare (Bundorf et al., 2024; McInerney et al., 2020), we matched treatment and comparison states on ACA Medicaid expansion status. Specifically, for each treatment state that expanded Medicaid, we selected comparison states that also expanded Medicaid within 1 year of the treatment state’s expansion date. We did not allow a state to contribute to the comparison group if it had an existing Medicare-Medicaid integration program (e.g., Minnesota Senior Health Options Program or Wisconsin Partnership Program) (Medicare Payment Advisory Commission, 2018) or enacted a change in Medicaid eligibility for older adult or disabled populations around the time of MMP implementation (e.g., as happened in Indiana in 2014) (Cornelio, 2023). Analyses using neighbor state comparisons included eight MMP states and 18 comparison states; Michigan was excluded due to the lack of suitable neighbor state comparisons. To account for age restrictions on MMP eligibility in certain states, analyses of Massachusetts and its comparison states were limited to beneficiaries under age 65, and analyses of South Carolina and its comparison states were limited to beneficiaries over age 65.

The second comparison group included Medicare beneficiaries who lived in counties without MMP availability within states that established MMPs *(within-state comparisons)*. This analysis included six states. Massachusetts, Rhode Island, and South Carolina were excluded because their MMP programs were statewide or near statewide. Treatment regions and both sets of comparison regions are mapped in Supplemental Appendix Figure 3.

Ohio made several changes to its Medicaid eligibility rules for older adults and disabled individuals 27 months after MMP rollout, resulting in an increase in Medicaid enrollment (Cornelio, 2023). To exclude the effects of this eligibility shift from our analyses, we restricted the Ohio post-period to 24 months in both the neighbor-state and within-state models.

### Outcomes

Our primary outcome was enrollment in full Medicaid, which we measured at the person-month-level in the MBSF. The denominator included person-months while an individual was alive and enrolled in Medicare in the study year. As a secondary outcome, we analyzed continuous enrollment in full Medicaid while alive (measured at the person-year level).

### Difference-in-Differences Models

We analyzed a 36-month window centered on MMP rollout. To account for heterogeneity across states, we first constructed state-specific estimates by fitting a separate DID model for each MMP state. For analyses using neighbor state comparisons, we estimated person-month-level linear models of the form:

$$\begin{array}{c} y_{ict}=\beta_{0}+\beta_{1}Post_{t}+\beta_{2}Post_{t}\times Trt_{c}+\;\beta_{3}X_{i}+\beta_{4}County_{c}\\ +\beta_{5}Year_{t}+\;\varepsilon_{ict} \end{array}$$

where *y_ict_* is a binary indicator of Medicaid enrollment for beneficiary *i* in county *c* and month *t*; *Post_t_* is an indicator of month *t* occurring on or after MMP rollout; and *Trt_c_* identifies treatment counties (i.e., whether an MMP was ever implemented in county *c* in the study window). We controlled for beneficiary demographics, denoted *X_i_*, (age, sex, race and ethnicity, and original reason for Medicare entitlement) and county and calendar year fixed effects, denoted $County_{c}$ and $Year_{t}$ , respectively. The county fixed effects subsume the main effect of $Trt_{c}$ . Because counties are nested within states, the county fixed effects also account for time-invariant state-level factors, such as state-specific Medicaid eligibility and renewal policies. The coefficient of interest, $\beta_{2}$ , captures the differential change in Medicaid enrollment before and after MMP rollout among low-income Medicare beneficiaries residing in counties with MMPs compared with beneficiaries in nearby regions without MMPs. We used a similar model to estimate state-specific treatment effects using within-state comparisons.

Next, we used a stacked DID model to pool these state-specific estimates into an overall estimate. In this specification, each set of treated and comparison counties that contribute to a state-specific estimate is analyzed as a sub-experiment (or “stack”), and treatment effects are aggregated across sub-experiments to yield a pooled estimate. This allowed us to obtain a pooled estimate accounting for the staggered timing of MMP rollouts across states. For analyses using neighbor state comparisons, this model had the form:

$$\begin{array}{c} y_{ict}=\beta_{0}+\beta_{1}Post_{t}+\beta_{2}Post_{t}\times Trt_{c}+\beta_{3}X_{i}+\beta_{4}\;County_{c}\\ \times stack_{m}+\beta_{5}Year_{t}\times stack_{m}+\;\varepsilon_{ict} \end{array}$$

We included county-by-stack fixed effects $\left(County_{c}\times stack_{m}\right)$ to account for counties potentially serving as comparisons across multiple sub-experiments and year-by-stack fixed effects ( $Year\times stack_{m}$ ) to control for time trends by sub-experiment. The coefficient $\beta_{2}$ is the pooled DID estimate. Finally, to obtain a pooled estimate for the analyses using within-state comparison counties, we ran the models:

$$\begin{array}{c} y_{ict}=\beta_{0}+\beta_{1}Post_{t}+\beta_{2}Post_{t}\times Trt_{c}+\beta_{3}X_{i}+\beta_{4}County_{c}\\ +\beta_{5}Year_{t}\times State_{s}+\;\varepsilon_{ict} \end{array}$$

Here, each sub-experiment includes counties within the same state. We include state-by-year fixed effects to control for time trends common to treatment and comparison counties within the same state.

DID assumes that time-varying factors unrelated to MMPs had similar effects on the treatment and comparison groups, making changes in the comparison group a plausible counterfactual for changes that would have been expected in the treatment group without the MMPs. To evaluate this assumption, we fitted event-study models to test for common (i.e., parallel) outcome trends in the pre-MMP period across treatment and comparison groups. Any evidence of differential pre-trends suggests that this DID assumption may not hold, underscoring a need for caution in interpreting estimates as causal effects of MMP implementation. In addition, to summarize baseline trend differences between the treatment and comparison groups, we fit a model on baseline data that included a linear time trend, treatment group indicator, and their interaction; the interaction captures the difference in outcome trends between groups before MMP implementation.

### Age Stratification

To account for differences between the older adult and disabled Medicare populations, we conducted stratified analyses for Medicare beneficiaries under age 65 versus age 65 and over. Because we restricted our sample to beneficiaries with at least 2 years of prior Medicare coverage, the second age stratum consisted of beneficiaries ages 67 and older. For simplicity, we refer to this second group as age 65 or older.

### Supplementary Analyses

We conducted four supplementary analyses. First, to examine the effects of MMP rollout on Medicaid retention, we analyzed continuous 12-month enrollment in full Medicaid while beneficiaries were alive. We indexed beneficiary-years relative to the month of MMP implementation. Because our measure of continuous enrollment could span two calendar years, we used the enhanced 5% Medicare sample, which maintains a consistent longitudinal sample. Second, we used the user-written Stata package HonestDiD to assess the sensitivity of our DID estimates to violations of the parallel trend assumption (Bravo et al., 2024; Rambachan & Roth, 2023). HonestDiD calculates confidence bounds for DID estimates under possible violations of the parallel trends assumption; these violations are modeled by varying the degree to which any differential pre-trends were assumed to continue post-MMP implementation. Third, we restricted our treatment sample to beneficiaries who resided in counties with MMP participation rates above the state-level median among demonstration counties (during the post-implementation period), as we expect any effect of the MMPs on Medicaid take-up to be concentrated in these counties. Fourth, we examined heterogeneity in the effects of MMP rollout on Medicaid enrollment in age subgroups of older adult Medicare beneficiaries (i.e., those ages 65–69, 70–74, 75–79, vs. ≥80 years) and among those with ≥1 versus 0 hospital admissions in the prior year (assessed from the Medicare Provider Analysis and Review file). Differential Medicaid take-up across groups related to age or prior use of care could affect the costs of covering marginal Medicaid enrollees.

## Results

### Descriptive Statistics

Table 2 displays characteristics of Medicare beneficiaries in the treatment and comparison groups during the baseline (pre-MMP) and post-MMP periods. In analyses of treatment states compared with neighbor states (labeled treatment group #1 vs. comparison group #1), a smaller proportion of beneficiaries in the treatment versus comparison groups was under age 65 at baseline (20.8% vs. 26.6%), a smaller proportion qualified for Medicare due to a disability (29.8% vs. 38.8%), and a larger proportion were Hispanic (28.3% vs. 5.7%). However, these differences changed minimally between the pre- and post-MMP periods. Constant differences between treatment and comparison groups are controlled for in the DID design. There were fewer imbalances in baseline characteristics in the analysis using a within-state comparison group (treatment group #2 vs. comparison group #2). For example, similar proportions of individuals in the treatment vs. within-state comparison groups were less than age 65 at baseline (20.1% vs. 20.3%), qualified for Medicare because of a disability (28.9% vs. 30.3%), and were Hispanic (28.3% vs. 27.4%). There were greater baseline differences in the proportions of White vs. Black beneficiaries, although these differences did not change appreciably between the baseline and post-MMP periods.

**Table 2.** Beneficiary Characteristics Across Study Groups and Periods, Among Residents of 20% Highest-Poverty Zip Codes.

	Treatment group #1:^a^ Beneficiaries in MMP counties		Comparison group #1:^b^ Beneficiaries in neighboring states without MMPs		Treatment group #2:^a^ Beneficiaries in MMP counties		Comparison group #2:^c^ Beneficiaries in non-MMP counties in same state	
Number or characteristic	*Pre-MMP* ^d^	*Post-MMP* ^e^	*Pre-MMP*	*Post-MMP*	*Pre-MMP*	*Post-MMP*	*Pre-MMP*	*Post-MMP*
*Number of beneficiary-months*	***11,569,107***	***12,247,929***	***16,380,937***	***16,325,356***	***11,155,445***	***11,737,230***	***5,711,152***	***6,060,671***
**Characteristic:**								
Age (% in category):								
<65 years	20.8	20.2	26.6	26.3	20.1	19.5	20.3	19.7
≥65 years	79.2	79.8	73.4	73.7	80.0	80.5	79.7	80.3
Female sex, %	55.4	54.9	54.8	54.4	55.6	55.1	53.5	53.2
Race and Ethnicity (% in category):^f^								
Non-Hispanic Black	26.8	25.7	25.3	26.1	29.5	28.2	11.9	11.9
Hispanic	28.3	30.7	5.7	6.4	28.3	30.8	27.4	29.2
Non-Hispanic White	36.8	34.6	65.6	63.7	33.9	31.7	54.6	52.4
Other	8.2	9.0	3.3	3.8	8.4	9.3	6.1	6.5
Original reason for Medicare eligibility (% in category):								
Age	69.1	68.7	60.3	59.6	69.9	69.6	68.8	68.5
Disability	29.8	30.2	38.8	39.5	28.9	29.2	30.3	30.7
End-stage renal disease	1.1	1.1	0.9	0.9	1.2	1.2	0.9	0.9

a Both treatment groups consist of Medicare beneficiaries residing in counties offering MMPs in Financial Alignment Demonstration states. Treatment group #1 excludes Michigan, which has no suitable neighbor comparison states. Treatment group #1 additionally restricts to beneficiaries aged <65 years in Massachusetts and ≥65 years in South Carolina, where age restrictions are placed on MMP eligibility. Treatment group #2 excludes Massachusetts, South Carolina, and Rhode Island, whose MMP programs are statewide or nearly statewide. ^b^Comparison group #1 includes Medicare beneficiaries living in non-MMP states neighboring states that implemented MMPs, matched on ACA Medicaid expansion status (see methods for details). Analyses using comparison group #1 restricts to beneficiaries aged <65 years in neighbor states of Massachusetts and ≥65 years in neighbor states of South Carolina due to age restrictions on MMP eligibility in those treatment states. ^c^Comparison group #2 includes Medicare beneficiaries living in non-MMP counties in Financial Alignment Demonstration states. Analyses using comparison group #2 exclude Massachusetts, South Carolina, and Rhode Island, whose MMP programs were statewide or nearly statewide. ^d^Pre-MMP implementation period is the 36 months prior to MMP implementation. ^e^Post-MMP implementation period is the 36 months following MMP implementation. ^f^Race and ethnicity assessed from enhanced RTI race/ethnicity variable in the Medicare Beneficiary Summary file. The RTI enhanced variable uses an imputation algorithm to identify additional Hispanic and Asian beneficiaries.

Supplemental Appendix Table 1 compares baseline characteristics of treated counties (e.g., number of physicians, hospitals, and nursing home beds per capita) to neighbor-state and within-state comparisons. In addition, Supplemental Appendix Table 2 compares pre- to post-MMP period changes in overall Medicare Advantage (MA) penetration rates. Between these periods, MA penetration rates increased by similar amounts in treatment and comparison regions.

Supplemental Appendix Table 1 also shows baseline monthly and continuous Medicaid enrollment rates for the treatment and comparison groups. Baseline enrollment rates varied across states, from 28.4% in treatment counties in Texas to 74.0% in treatment counties in Massachusetts for beneficiaries under age 65, and from 12.0% for treatment counties in Virginia to 39.4% for treatment counties in California for beneficiaries over age 65. These rates do not represent take-up among eligible individuals because we cannot measure Medicaid eligibility directly.

### Primary Difference-in-Differences Estimates

**Figure 1.**
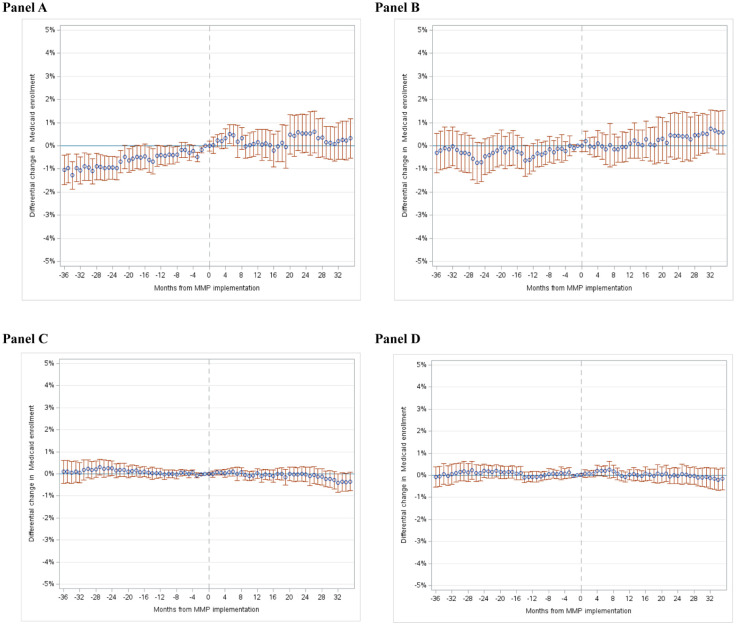
Event-Study Estimates of Monthly Medicaid Enrollment, Among Residents of 20% Highest-Poverty Zip Codes. Panel A (Beneficiaries under age 65, treatment group vs. comparison group #1), Panel B (Beneficiaries under age 65, treatment group vs. comparison group #2), Panel C (Beneficiaries aged 65 and older, treatment group vs. comparison group #1), and Panel D (Beneficiaries aged 65 and older, treatment group vs. comparison group #2). *Note.* Estimates are among Medicare beneficiaries living in the 20% of zip codes with the highest poverty rates among individuals ages 65 and older. Figures depict event-study estimates of the differential change in enrollment in full Medicaid between the treatment group (residents of counties with MMPs in Financial Alignment Demonstration states) versus comparison groups. The outcome is a binary person-month-level indicator for enrollment in full Medicaid in months when a beneficiary was alive and enrolled in Medicare. Comparison group #1 includes Medicare beneficiaries living in non-MMP states neighboring those that implemented MMPs, matched on ACA Medicaid expansion status (see Table 1). Comparison group #2 includes Medicare beneficiaries living in counties of states where MMPs were not implemented (limited to 6 states where MMPs were implemented in a subset of counties). Event-study estimates obtained from a beneficiary-month-level linear regression model predicting Medicaid enrollment as a function of treatment state indicators, event-time, and the interaction of these terms. Estimates adjusted for sex, race and ethnicity, and original reason for Medicare eligibility. 95% confidence intervals constructed using standard errors clustered by county. Panels A-B are among beneficiaries under age 65 and Panels C-D are among beneficiaries ages 65 and older.

Table 3 summarizes DID estimates for changes in monthly Medicaid enrollment using neighbor-state comparisons (left columns) and within-state comparisons (right columns). The top panels show our estimates for beneficiaries under age 65, and the bottom panels are for beneficiaries over age 65. Alongside each set of DID estimates, we test for differential pre-MMP trends between the treatment vs. comparison groups; these trends are also visualized in the event-study plots in Figure 1.

**Table 3.** Difference-in-Differences Estimates of Monthly Medicaid Enrollment, Among Residents of 20% Highest-Poverty Zip Codes.

Panel A. Monthly Medicaid enrollment, among beneficiaries < 65 years								
	Treatment group vs. comparison group #1 ^a^				Treatment group vs. comparison group #2 ^b^			
	Differential trend pre-MMP implementation^c^		DID estimate, post vs. pre-MMP implementation		Differential trend pre-MMP implementation^c^		DID estimate, post vs. pre-MMP implementation	
DID Estimates	*Estimate, percentage points (pp)*	*95% CI*	*Estimate, pp*	*95% CI*	*Estimate, pp*	*95% CI*	*Estimate, pp*	*95% CI*
Pooled DID estimate^d^	0.36	0.13, 0.59	0.85	0.18, 1.52	0.05	−0.25, 0.36	0.50	−0.11, 1.11
State-specific estimates:^e^								
California	−0.15	−0.56, 0.26	1.59	−0.15, 3.33	−0.13	−0.65, 0.38	−0.15	−1.25, 0.96
Illinois	0.88	0.55, 1.20	0.90	0.23, 1.57	−0.05	−1.10, 1.21	0.96	−1.65, 3.58
Massachusetts	0.99	0.64, 1.33	3.21	2.15, 4.27				
Michigan					0.42	−0.75, 1.59	1.94	−0.53, 4.41
Ohio	0.68	0.23, 1.13	−1.05	−2.00, −0.10	0.53	−0.43, 1.49	1.59	−0.07, 3.24
Rhode Island	−0.57	−1.38, 0.25	6.16	4.50, 7.82				
South Carolina								
Texas	−0.22	−0.65, 0.21	−1.07	−2.03, −0.10	−0.02	−0.51, 0.47	0.44	−0.57, 1.45
Virginia	0.57	−0.13, 1.28	1.23	−0.04, 2.50	0.09	−0.67, 0.86	1.06	−0.47, 2.59
Panel B. Monthly Medicaid enrollment, among beneficiaries ≥ 65 years								
	Treatment group vs. comparison group #1 ^a^				Treatment group vs. comparison group #2 ^b^			
	Differential trend pre-MMP implementation^c^		DID estimate, post vs. pre-MMP implementation		Differential trend pre-MMP implementation^c^		DID estimate, post vs. pre-MMP implementation	
DID Estimates	*Estimate, pp*	*95% CI*	*Estimate, pp*	*95% CI*	*Estimate, pp*	*95% CI*	*Estimate, pp*	*95% CI*
Pooled DID estimate^d^	−0.08	−0.25, −0.10	−0.16	−0.58, 0.25	−0.02	−0.18, 0.13	−0.05	−0.43, 0.33
State-specific estimates:^e^								
California	−0.24	−0.52, 0.04	−0.76	−1.51, −0.01	−0.18	−0.39, 0.04	−0.52	−0.91, −0.13
Illinois	0.76	0.64, 0.88	1.63	1.31, 1.95	0.48	0.19, 0.76	0.93	0.28, 1.59
Massachusetts								
Michigan					0.22	−0.17, 0.62	1.39	0.32, 2.46
Ohio	0.10	−0.10, 0.30	0.23	−0.39, 0.84	0.10	−0.17, 0.36	0.54	0.01, 1.06
Rhode Island	0.77	0.31, 1.24	3.96	2.66, 5.25				
South Carolina	−0.17	−0.43, 0.10	−0.10	−0.55, 0.35				
Texas	−0.44	−0.75, −0.12	−1.29	−2.08, −0.49	0.01	−0.28, 0.30	0.04	−0.76, 0.83
Virginia	−0.11	−0.37, 0.15	0.44	−0.06, 0.93	−0.23	−0.68, 0.22	0.03	−0.86, 0.93

*Note.* Difference-in-differences (DID) estimates reflect differential change in enrollment in full Medicaid between the treatment groups (residents of counties with MMPs in Financial Alignment Demonstration States) vs. comparison groups for these states (see methods for details). The outcome is a binary person-month-level indicator for enrollment in full Medicaid in months when a beneficiary was alive and enrolled in Medicare. Estimates are in percentage points (pp). Estimates were obtained from a beneficiary-month-level linear regression model predicting Medicaid enrollment as a function of treatment state indicators, event-time, and the interaction of these terms. Estimates are adjusted for sex, race and ethnicity, and original reason for Medicare eligibility. 95% confidence intervals constructed using standard errors clustered by county. Shaded cells denote where analyses were not conducted because the MMP was not implemented in the age group shown (e.g., Massachusetts’ MMP for beneficiaries ages ≥65 years) or because a comparison group was not available.

a Comparison group #1 includes Medicare beneficiaries living in non-MMP states neighboring states that implemented MMPs, matched on ACA Medicaid expansion status (see methods for details). ^b^Comparison group #2 includes Medicare beneficiaries living in non-MMP counties in Financial Alignment Demonstration states. This analysis excludes Massachusetts, South Carolina, and Rhode Island, whose MMP programs are statewide or nearly statewide. ^c^Differential pre-MMP implementation trend reported as the annual average differential change in enrollment in full Medicaid between treatment and comparison groups during the 3-year period prior to MMP implementation. ^d^Pooled difference-in-differences estimates aggregate over the state-specific estimates using the method of stacked difference-in-differences. ^e^State-specific estimates obtained from separate models fitted on observations from each state and comparison group. See Supplemental Appendix Figure 4 for event-study plots depicting monthly differential changes in Medicaid enrollment analyzed separately by treatment state.

Among beneficiaries under age 65, the pooled DID estimate using the neighbor-state comparison group was positive and statistically significant (0.85 percentage points; 95% CI: 0.18, 1.52), indicating a differential increase in Medicaid enrollment (**Panel A of** Table 3). However, as shown in the event-study analysis (**Panel A of** Figure 1), in the under-65 population, Medicaid enrollment during the baseline period increased differentially in treatment vs. neighbor states (pre-trend difference: 0.36 percentage points per beneficiary-year; 95% CI: 0.13, 0.59). Given this pre-trend difference, we cannot interpret the DID estimate as a causal effect of MMP implementation. State-specific DID estimates for Illinois and Massachusetts were positive and statistically significant, although both states had similarly significant differences in pre-trends (see Supplemental Appendix Figure 4a-4c for event-study analyses by state). In Rhode Island, differential increases in Medicaid enrollment emerged 18 months after the state’s MMP started. Because of the delayed change, there is substantial uncertainty about whether the increase was due to MMP implementation. Analyses using within-state comparison groups found no differential change in monthly Medicaid enrollment in the pooled analyses and in all six state-specific analyses. The event-study analyses (**Panel B of** Figure 1 for the pooled analysis and Supplemental Appendix Figure 4d-4f for analyses by state) confirmed these findings.

Among beneficiaries over age 65, there was no differential change in monthly Medicaid enrollment in treated states (pooled) vs. neighbor-state and within-state comparisons (**Panel B of** Table 3). Pooled event study analyses (**Panels C and D of** Figure 1) show estimates near zero that are precisely estimated, thus ruling out even small positive or negative differential enrollment changes. In state-specific analyses, we found a differential enrollment increase in Rhode Island 18 months post-MMP implementation, and modest differential increases in Illinois, and for the within-state models, Michigan and Ohio. However, the enrollment increase in Rhode Island was not likely related to the MMP, as this change occurred well after the program’s implementation. For Illinois, there were significant differences in pre-trends in analyses that used neighbor-state (Supplemental Appendix Figure 4a) and within-state (Supplemental Appendix Figure 4d) comparisons. There were also statistically insignificant, though still visible, pre-trend differences in Michigan relative to within-state comparisons (Supplemental Appendix Figure 4e).

### Supplementary Analyses

Results were similar for the probability of continuous, 12-month Medicaid enrollment (Supplemental Appendix Table 3 and Supplemental Appendix Figure 5). In pooled analyses among beneficiaries under age 65, we found no differential change in continuous enrollment using neighbor-state comparisons (DID estimate: 0.64 percentage points; 95% CI: -0.23, 1.50) and within-state comparisons (DID estimate: 0.32 percentage points; 95% CI: -0.60, 1.24). Pooled analyses for beneficiaries over age 65 also identified no differential change in continuous Medicaid enrollment. State-specific event study estimates for continuous Medicaid enrollment are in Supplemental Appendix Figure 6.

Sensitivity analyses revealed that none of the pooled DID estimates were statistically significant across scenarios that modeled different possible violations of the parallel trends assumption (Supplemental Appendix Figure 7). Subgroup analyses did not reveal differential increases in monthly Medicaid enrollment among age subgroups of older adult beneficiaries. Although we found a differential increase in Medicaid enrollment among beneficiaries under age 65 without a hospital admission in the prior 12 months, pre-trends differed significantly in the analyses using neighbor-state comparisons (Supplemental Appendix Table 4). In sensitivity analyses restricting the treatment group to residents of counties with above-median MMP participation rates (based on the distributions shown in Supplemental Appendix Figure 8), no differential changes were found in monthly or continuous Medicaid enrollment (Supplemental Appendix Table 5).

## Discussion

This study examined whether the rollout of MMPs—one of the largest multi-state efforts to test ICPs—was associated with changes in Medicaid enrollment and retention among Medicare beneficiaries residing in communities with a high concentration of low-income older adults. Overall, we found no evidence that the rollout of MMPs led to increases in monthly Medicaid enrollment or continuous Medicaid coverage, relative to changes in comparison regions where MMPs were not implemented. Although analyses of some states and the under-65 population found a modest differential increase in Medicaid enrollment post-MMP implementation, these changes typically followed a trend of rising Medicaid enrollment preceding MMP implementation in treatment vs. comparison regions. Thus, it is unlikely that continued changes in Medicaid enrollment were the result of MMP implementation. We also found no evidence that Medicaid enrollment increased differentially in analyses of subpopulations expected to cost more due to age or prior health care use.

These findings suggest that the expansion of ICPs alone may not materially increase Medicaid enrollment rates. However, targeted policies that help Medicare beneficiaries maintain Medicaid enrollment could complement and enhance Medicare-Medicaid integration efforts. Disenrollment from Medicaid can erode opportunities to integrate coverage because loss of Medicaid—and thus dual-eligibility status—requires individuals to disenroll from ICPs. Reducing Medicaid churn can prevent these disruptions, preserving enrollee access to ICPs’ care coordinators and in-network providers. It also offers plans a more stable enrollee base, which could improve the financial viability of ICPs and incentivize long-term investments in care management, especially when plans expect to retain enrollees over time. Sable Medicaid enrollment also ensures that low-income Medicare beneficiaries receive cost-sharing assistance for medical services and maintain access to long-term care services, including and home- and community-based services.

To our knowledge, this is the first study to evaluate whether Medicaid enrollment is affected by policies designed to integrate coverage for dual-eligible beneficiaries. However, several recent studies have examined the effects of other policies on Medicaid take-up among low-income Medicare beneficiaries. For example, McInerny, Mellor, and Sabik used U.S. Census Bureau data to study the effects of ACA Medicaid expansions to working-age adults (ages 19–64 years) on Medicaid take-up among low-income adults ages 65 and older (McInerney et al., 2020). They found that exposure to ACA Medicaid expansions among low-income adults approaching age 65 increased their likelihood of enrolling in Medicaid past 65 by approximately 2 percentage points. These effects were driven, in part, by pre-65 Medicaid coverage increasing the likelihood that individuals maintain Medicaid after aging into Medicare (termed an “on-ramp” effect). A more recent study using administrative data found that exposure to ACA Medicaid expansions before age 65 led to an even larger (9 percentage point) increase in Medicaid enrollment after individuals had entered Medicare (Bundorf et al., 2024). Other analyses found that Medicaid enrollment among low-income Medicare beneficiaries was higher, and churn was lower, in states that automatically enrolled individuals receiving Supplemental Security Income in Medicaid (Burns et al., 2012; Cornelio, 2023; Roberts et al., 2019). Together, these studies highlight how policies that simplify Medicaid enrollment and provide an on-ramp to Medicaid coverage (e.g., for individuals newly eligible for Medicare and Medicaid) can result in meaningful increases in Medicaid take-up.

### Limitations

This study had several limitations. First, unmeasured time-varying factors at the county or beneficiary levels could have biased our DID estimates. To reduce bias, we matched treatment and comparison regions to account for concurrent effects of ACA Medicaid expansions. However, bias could still arise if the effects of ACA expansions or other time-dependent factors differed across treatment and comparison counties. Second, we could not directly identify Medicaid-eligible but unenrolled individuals. Instead, we used zip codes with high-poverty rates among older adults to identify a population with a higher likelihood of Medicaid eligibility. Third, our estimates rely on an intention-to-treat design that compares changes among beneficiaries in counties with MMPs—regardless of individual-level MMP enrollment—to those in comparison counties without MMPs. While this approach avoids bias from comparing voluntary enrollees to non-enrollees, it may dilute the estimated effect of MMPs on Medicaid enrollment by averaging outcomes across both groups. However, our findings were similar when we limited the analysis to counties with MMP penetration rates above the state median, where such attenuation bias would likely be smaller. Fourth, we focused on implementation of MMPs to capitalize on a natural experiment arising from state variation in the rollout of these programs. However, MMPs are temporary demonstration plans, which are scheduled to conclude in 2025, and our findings may not generalize to other, ongoing categories of ICPs.

## Conclusion

In conclusion, this study found that implementation of MMPs—financially integrated managed care plans for dual-eligible beneficiaries—did not lead to increases in monthly or continuous annual Medicaid enrollment among Medicare beneficiaries residing in high-poverty zip codes. Dedicated policy reforms to increase Medicaid take-up and retention could complement efforts to expand integrated care plans for dual-eligible beneficiaries.

## Supplemental Material

sj-pdf-1-mcr-10.1177_10775587251372267 – Supplemental material for Effects of Dual-Eligible Integrated Care Plans on Medicaid Enrollment and Retention: Evidence From the Implementation of Medicare-Medicaid Plans Supplemental material, sj-pdf-1-mcr-10.1177_10775587251372267 for Effects of Dual-Eligible Integrated Care Plans on Medicaid Enrollment and Retention: Evidence From the Implementation of Medicare-Medicaid Plans by Eric T. Roberts, Eliza Macneal, Kenton J. Johnston and José F. Figueroa in Medical Care Research and Review

## References

[bibr1-10775587251372267] Allen S. M. Piette E. R. Mor V. (2014). The adverse consequences of unmet need among older persons living in the community: Dual-eligible versus Medicare-only beneficiaries. The Journals of Gerontology: Series B, 69(Suppl_1), S51–S58. 10.1093/geronb/gbu124 PMC4303067 25342823

[bibr2-10775587251372267] Barnette L. P. (2021, June 15). CMS update on Medicare A/B payments for New York FIDA-IDD MMP. Centers for Medicare and Medicaid Services. https://www.cms.gov/files/document/cmsupdateoncy2021medicareabpaymentsforphp.pdf

[bibr3-10775587251372267] Bella M. Hinckley J. Roberts E. Weil A. Weiner J. Werner R. M. (2024). Forging a path toward integrated care for dually eligible individuals. https://d197nivf0nbma8.cloudfront.net/uploads/2024/11/V3_Forging-a-Path-Toward-Integrated-Care-for-Dually-Eligible-Individuals_Final-for-Webv3.pdf

[bibr4-10775587251372267] Bravo M. C. Roth J. Vilhuber L. (2024). Mcaceresb/stata-honestdid. https://github.com/mcaceresb/stata-honestdid (Original work published 2022)

[bibr5-10775587251372267] Bundorf M. K. McInerney M. Simon K. Winecoff R. (2024). Spillovers in public benefit enrollment: How does expanding public health insurance for working-age adults affect future health insurance choices? (Working paper no. 32675). National Bureau of Economic Research. 10.3386/w32675

[bibr6-10775587251372267] Burns A. Peña M. T. Mohamed M. Freed M. Watts M. O. (2024, October 22). What are the primary Medicaid eligibility pathways for dual-eligible individuals? KFF. https://www.kff.org/medicaid/issue-brief/what-are-the-primary-medicaid-eligibility-pathways-for-dual-eligible-individuals/

[bibr7-10775587251372267] Burns M. E. O’Hara B. J. Huskamp H. A. Soumerai S. B. (2012). Uninsurance and its correlates among poor adults with disabilities. Journal of Health Care for the Poor and Underserved, 23(4), 1630–1646. 23698677 10.1353/hpu.2012.0197 PMC3671490

[bibr8-10775587251372267] Centers for Medicare and Medicaid Services. (n.d.-a). Financial alignment initiative for Medicare-Medicaid enrollees. CMS.gov. https://www.cms.gov/priorities/innovation/innovation-models/financial-alignment

[bibr9-10775587251372267] Centers for Medicare and Medicaid Services. (2019, March 19). Joint rate setting process for the financial alignment initiative’s capitated model. CMS. https://www.cms.gov/files/document/capitatedmodelratesettingprocess03192019.pdf

[bibr10-10775587251372267] Cornelio N. (2023). Eligibility and enrollment in Medicaid for low-income Medicare beneficiaries: The role of state policy [Unpublished doctoral dissertation]. University of Pittsburgh. https://d-scholarship.pitt.edu/44025/

[bibr11-10775587251372267] Feng Z. Vadnais A. Vreeland E. Haber S. Wiener J. Baker B. (2019a, May 8). Analysis of pathways to dual eligible status: Final report. Office of the Assistant Secretary for Planning and Evaluation. https://aspe.hhs.gov/reports/analysis-pathways-dual-eligible-status-final-report-0

[bibr12-10775587251372267] Feng Z. Vadnais A. Vreeland E. Segelman M. Ferrell A. Wiener J. M. Baker B. (2019b, May 8). Loss of Medicare-Medicaid dual eligible status: Frequency, contributing factors and implications. Office of the Assistant Secretary for Planning and Evaluation. https://aspe.hhs.gov/reports/loss-medicare-medicaid-dual-eligible-status-frequency-contributing-factors-implications

[bibr13-10775587251372267] Garrow R. Mellor J. M. McInerney M. Sabik L. M. (2023). Examining Medicaid participation and Medicaid entry among senior Medicare beneficiaries with linked administrative and survey data. Medical Care Research and Review: MCRR, 80(1), 109–125. 10.1177/10775587221101297 35730585 PMC11867305

[bibr14-10775587251372267] Grabowski D. C. (2007). Medicare and Medicaid: Conflicting incentives for long-term care. The Milbank Quarterly, 85(4), 579–610. 10.1111/j.1468-0009.2007.00502.x 18070331 PMC2690349

[bibr15-10775587251372267] Grabowski D. C. Joyce N. R. McGuire T. G. Frank R. G. (2017). Passive enrollment of dual-eligible beneficiaries into Medicare and Medicaid managed care has not met expectations. Health Affairs, 36(5), 846–854. 10.1377/hlthaff.2016.1082 28461351 PMC5665685

[bibr16-10775587251372267] Greene A. (2017, March). Beneficiary experience: Early findings from focus groups with enrollees participating in the financial alignment initiative. Centers for Medicare and Medicaid Services. https://www.cms.gov/medicare-medicaid-coordination/medicare-and-medicaid-coordination/medicare-medicaid-coordination-office/financialalignmentinitiative/downloads/focusgroupissuebrief508032017.pdf

[bibr17-10775587251372267] Johnston K. J. Roberts E. T. Figueroa J. F. (2024). Helping dual-eligible individuals navigate Medicare and Medicaid. Journal of the American Medical Association, 332, 1971–1972. 10.1001/jama.2024.20355 39412776

[bibr18-10775587251372267] Keohane L. M. Trivedi A. N. Mor V. (2017). The role of Medicare’s inpatient cost-sharing in Medicaid entry. Health Services Research, 53(2), 711–729. 10.1111/1475-6773.12682 28295261 PMC5867186

[bibr19-10775587251372267] Kyle M. A. Frakt A. B. (2021). Patient administrative burden in the U.S. health care system. Health Services Research, 56(5), 755–765. 10.1111/1475-6773.13861 34498259 PMC8522562

[bibr20-10775587251372267] Lakhmani E. W. Lomas A. Wood E. (2022, March). Preventing and addressing unnecessary Medicaid eligibility churn among dually eligible individuals: Opportunities for states. Integrated Care Resource Center. https://www.integratedcareresourcecenter.com/sites/default/files/ICRC_Addressing_Medicaid_Churn.pdf

[bibr21-10775587251372267] Ma Y. Roberts E. T. Johnston K. J. Orav E. J. Figueroa J. F. (2024). Medicaid eligibility loss among dual-eligible beneficiaries before and during COVID-19 public health emergency. Journal of the American Medical Association Network Open, 7(4), Article e245876. 10.1001/jamanetworkopen.2024.5876 PMC11009828 38602676

[bibr22-10775587251372267] McInerney M. Mellor J. M. Sabik L. M. (2020). Welcome mats and on-ramps for older adults: The impact of the affordable care act’s Medicaid expansions on dual enrollment in Medicare and Medicaid. Journal of Policy Analysis and Management : [The Journal of the Association for Public Policy Analysis and Management], 40(1), Article 12. 10.1002/pam.22259 PMC8238124 34194129

[bibr23-10775587251372267] Medicaid and CHIP Payment and Access Commission. (2021, January 28). Establishing a unified program for dually eligible beneficiaries design considerations. https://www.macpac.gov/publication/establishing-a-unified-program-for-dually-eligible-beneficiaries-design-considerations/

[bibr24-10775587251372267] Medicaid and CHIP Payment and Access Commission. (2022a, June 15). Raising the bar: Requiring state integrated care strategies. https://www.macpac.gov/publication/raising-the-bar-requiring-state-integrated-care-strategies/

[bibr25-10775587251372267] Medicaid and CHIP Payment and Access Commission. (2022b, October 17). Inventory of evaluations of integrated care programs for dually eligible beneficiaries. https://www.macpac.gov/publication/inventory-of-evaluations-of-integrated-care-programs-for-dually-eligible-beneficiaries/

[bibr26-10775587251372267] Medicare Payment Advisory Commission. (2016, June). Issues affecting dual-eligible beneficiaries: CMS’s financial alignment demonstration and the Medicare Savings Programs. https://www.medpac.gov/wp-content/uploads/import_data/scrape_files/docs/default-source/reports/chapter-9-issues-affecting-dual-eligible-beneficiaries-cms-s-financial-alignment-demonstration-and-t.pdf

[bibr27-10775587251372267] Medicare Payment Advisory Commission. (2018, June). Managed care plans for dual-eligible beneficiaries. https://www.medpac.gov/wp-content/uploads/import_data/scrape_files/docs/default-source/reports/jun18_ch9_medpacreport_sec.pdf

[bibr28-10775587251372267] Medicare Payment Advisory Commission. (2019, June). Promoting integration in dual-eligible special needs plans. https://www.medpac.gov/wp-content/uploads/import_data/scrape_files/docs/default-source/reports/jun19_ch12_medpac_reporttocongress_sec.pdf

[bibr29-10775587251372267] Medicare Payment Advisory Commission & Medicaid and CHIP Payment and Access Commission. (2024, January 22). Data book: Beneficiaries dually eligible for Medicare and Medicaid. https://www.macpac.gov/publication/data-book-beneficiaries-dually-eligible-for-medicare-and-medicaid-3/

[bibr30-10775587251372267] Mellor J. M. Cunningham P. J. Britton E. Behrens M. Urmi A. F. Vega V. (2024). Beneficiary experience of care by level of integration in dual eligible special needs plans. Journal of the American Medical Association Health Forum, 5(6), Article e241383. 10.1001/jamahealthforum.2024.1383 PMC11161838 38848088

[bibr31-10775587251372267] Office of Management and Budget. (2021, July). Study to identify methods to assess equity: Report to the president. National Archives. https://bidenwhitehouse.archives.gov/wp-content/uploads/2021/08/OMB-Report-on-E013985-Implementation_508-Compliant-Secure-v1.1.pdf

[bibr32-10775587251372267] Peña M. T. Mohamed M. Biniek J. F. Burns A. Cubanski J. Neuman T. (2024, October). Landscape of Medicare and Medicaid coverage arrangements for dual-eligible individuals across states. https://www.kff.org/medicare/issue-brief/the-landscape-of-medicare-and-medicaid-coverage-arrangements-for-dual-eligible-individuals-across-states/

[bibr33-10775587251372267] Pezzin L. E. Kasper J. D. (2002). Medicaid enrollment among elderly Medicare beneficiaries: Individual determinants, effects of state policy, and impact on service use. Health Services Research, 37(4), 827–847. 10.1034/j.1600-0560.2002.55.x 12236387 PMC1464012

[bibr34-10775587251372267] Rambachan A. Roth J. (2023). A more credible approach to parallel trends. The Review of Economic Studies, 90(5), 2555–2591. 10.1093/restud/rdad018

[bibr35-10775587251372267] Riley G. F. Zhao L. Tilahun N. (2014). Understanding factors associated with loss of Medicaid coverage among dual eligibles can help identify vulnerable enrollees. Health Affairs, 33(1), 147–152. 10.1377/hlthaff.2013.0396 24395947

[bibr36-10775587251372267] Roberts E. T. Duggan C. Stein R. Jonnadula S. Johnston K. J. Figueroa J. F. (2024). Quality, spending, utilization, and outcomes among dual-eligible Medicare-Medicaid beneficiaries in integrated care programs: A systematic review. Journal of the American Medical Association Health Forum, 5(7), Article e242187. 10.1001/jamahealthforum.2024.2187 PMC11259897 39028653

[bibr37-10775587251372267] Roberts E. T. Welsh J. H. Donohue J. M. Sabik L. M. (2019). Association of state policies with Medicaid disenrollment among low-income Medicare beneficiaries. Health Affairs, 38(7), 1153–1162. 10.1377/hlthaff.2018.05165 31260363 PMC6690844

[bibr38-10775587251372267] Sen Cassidy B. [R-LA]. (2024, March 14). S.3950—118th Congress (2023-2024): DUALS Act of 2024 [Legislation]. https://www.congress.gov/bill/118th-congress/senate-bill/3950

[bibr39-10775587251372267] Spillman B. Waidmann T. (2014, May 31). Rates and timing of Medicaid enrollment among older Americans. Office of the Assistant Secretary for Planning and Evaluation. https://aspe.hhs.gov/reports/rates-timing-medicaid-enrollment-among-older-americans

[bibr40-10775587251372267] Tjhia R. Lapedis J. Roberts E. T. Tipirneni R. (2024). Older adults and people with disabilities are at risk for Medicaid disenrollment. Journal of the American Geriatrics Society, 72(7), 2274–2277. 10.1111/jgs.18834 38433713 PMC11701976

[bibr41-10775587251372267] Velasquez D. E. Orav E. J. Figueroa J. F. (2023). Enrollment and characteristics of dual-eligible Medicare and Medicaid beneficiaries in integrated care programs. Health Affairs, 42(5), 683–692. 10.1377/hlthaff.2022.01321 37126757

[bibr42-10775587251372267] Wolff J. L. Nicholas L. H. Willink A. Mulcahy J. Davis K. Kasper J. D. (2019). Medicare spending and the adequacy of support with daily activities in community-living older adults with disability: An observational study. Annals of Internal Medicine, 170(12), 837–844. 10.7326/M18-2467 31132789 PMC6736697

