# Supplementary material for: Effects of Dual-Eligible Integrated Care Plans on Medicaid Enrollment and Retention: Evidence From the Implementation of Medicare-Medicaid Plans
Source: Med Care Res Rev. 2025 Oct 2;83(1):20–32. doi: 10.1177/10775587251372267 (PMC12759090; doi:10.1177/10775587251372267)
Supplement: sj-pdf-1-mcr-10.1177_10775587251372267 – Supplemental material for Effects of Dual-Eligible Integrated Care Plans on Medicaid Enrollment and Retention: Evidence From the Implementation of Medicare-Medicaid Plans [file sj-pdf-1-mcr-10.1177_10775587251372267.pdf]

TABLE OF CONTENTS

Appendix Figure 1: Flow diagram of sample inclusion – *p. 2*

Appendix Table 1: Baseline area-level characteristics of treatment and comparison counties – *pp. 3–4*

Appendix Table 2: Differential change in Medicare Advantage penetration rates between MMP counties and two regional comparison groups – *p. 5*

Appendix Figure 2: MMP enrollment rates in demonstration counties of nine analyzed Financial Alignment Initiative states – *p. 6*

Appendix Figure 3: Map of MMP treatment counties and neighbor-state and within-state comparison counties – *p. 7*

Appendix Figure 4: Event-study estimates of monthly Medicaid enrollment, stratified by MMP state – *pp. 8-13*

Appendix Table 3: Difference-in-differences estimates of continuous Medicaid enrollment – *pp. 14–15*

Appendix Figure 5: Event-study estimates of continuous annual Medicaid enrollment – *pp. 16–17*

Appendix Figure 6: Event-study estimates of continuous annual Medicaid enrollment, stratified by MMP state – *pp. 18–23*

Appendix Figure 7: Sensitivity analysis: confidence intervals with extrapolation of trend violations – *pp. 24–25*

Appendix Table 4. Heterogeneity of difference-in-differences estimates of monthly Medicaid enrollment – *pp. 26–27*

Appendix Figure 8: Within-state treatment county level median MMP enrollment rate in the 36 months following MMP implementation – *p. 28*

Appendix Table 5: Difference-in-differences estimates limited to high MMP penetration counties – *pp. 29–30*

Appendix Figure 1: Flow diagram of sample inclusion

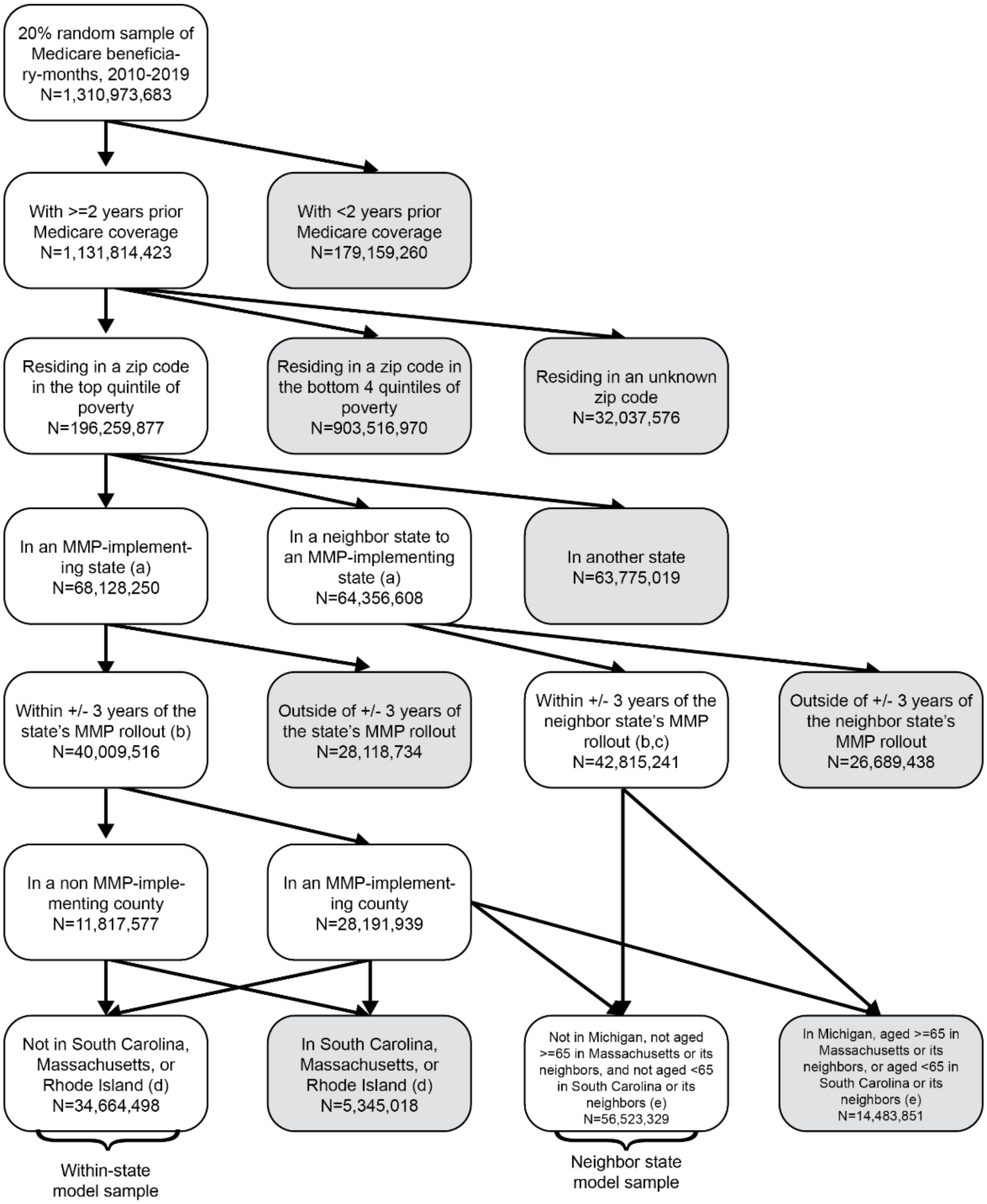

(a) See the 9 analyzed MMP-implementing states and their neighbor comparison states in Table 1.
(b) In Ohio and its neighboring states, the study period is capped at 2 (rather than 3) years after MMP rollout due to a Medicaid policy change in the third year after the MMP rollout.
(c) About 5 million person-months in Kentucky and North Carolina appear twice in the sample as neighbor controls for two separate MMP states. As a result, the sum of Ns at this juncture exceeds the N of the parent cell by 5 million.
(d) South Carolina, Massachusetts, and Rhode Island were excluded from the within-state model analysis. See methods for details.
(e) Michigan, >=65-year-olds in Massachusetts and its neighbors, and <65-year-olds in South Carolina and its neighbors were excluded from neighbor state model analysis. See methods for details.

Appendix Table 1: Baseline area-level characteristics of treatment and comparison counties **Panel A:** Counties implementing MMPs vs. neighbor-state comparison counties (comparison group #1)

**MMP implementation state:**		**California**		**Illinois**		**Massachusetts**		**Ohio**		**Rhode Island**		**South Carolina**		**Texas**		**Virginia**	
	Data year	T (N=7)	C (N=68)	T (N=21)	C (N=291)	T (N=12)	C (N=94)	T (N=29)	C (N=242)	T (N=5)	C (N=8)	T (N=44)	C (N=259)	T (N=6)	C (N=379)	T (N=102)	C (N=195)
Monthly Medicaid enrollment rate, sample beneficiaries age <65	3 years prior to MMP roll-out	60.6	42.4	59.4	27.9	74.0	54.1	39.1	37.7	55.8	55.0			28.4	37.7	30.5	39.7
Monthly Medicaid enrollment rate, sample beneficiaries age ≥65		39.4	19.6	24.5	11.8			14.1	17.1	29.0	24.6	17.9	13.9	18.7	14.2	12.0	15.4
Continuous annual Medicaid enrollment rate, sample beneficiaries age <65	3 years prior to MMP roll-out	55.7	39.5	57.1	27.1	69.9	49.2	31.3	35.7	51.1	49.3			25.4	36.2	28.5	38.2
Continuous annual Medicaid enrollment rate, sample beneficiaries age ≥65		35.7	17.7	23.2	11.5			11.7	16.2	28.2	24.0	16.6	13.6	17.7	13.5	11.3	15.0
Percent MA penetration (mean)	2015	48.8	24.6	25.3	18.1	18.7	27.1	43.5	28.5	33.8	24.5	24.4	28.5	42.1	12.0	16.9	28.1
Percent in poverty (mean)	2015	13.6	16.6	12.9	17.0	12.2	13.0	12.4	18.6	11.2	9.6	21.2	20.6	19.1	17.7	13.8	18.8
Percent White (mean)	2010	38.7	74.1	80.6	92.8	80.0	85.5	85.8	92.4	86.5	78.6	56.7	66.0	28.2	78.7	69.5	78.2
Percent Black (mean)	2010	4.8	1.2	7.8	2.4	5.1	4.5	7.9	3.4	2.7	6.2	36.6	24.9	10.8	11.0	21.2	13.9
Percent Hispanic (mean)	2010	37.2	16.2	7.2	2.9	8.3	5.8	2.9	2.2	6.1	9.9	4.2	6.0	56.2	5.1	4.9	4.8
Rural county (%)	2013	0.0	88.2	76.2	93.6	50.0	73.4	51.7	88.4	0.0	62.5	97.7	84.2	33.3	91.3	56.3	85.1
N short term gen hospital beds per 1000 residents (mean)	2015	1.71	1.63	2.44	2.48	1.93	2.36	2.06	2.50	1.43	1.58	2.01	2.17	2.52	4.14	2.10	1.92
N nursing home beds per 1000 residents (mean)	2015	0.08	0.16	0.18	0.38	0.08	0.52	0.08	0.30	0.00	0.01	0.14	0.49	0.11	1.07	0.08	0.19
Physicians per 1000 residents (mean)	2015	2.95	1.34	2.05	0.90	4.15	2.35	2.21	1.34	3.50	2.82	1.32	1.19	2.37	0.79	1.94	1.29

Panel B: Counties implementing MMPs vs. within-state comparison counties (comparison group #2)

**MMP implementation state:**		**California**		**Illinois**		**Michigan**		**Ohio**		**Texas**		**Virginia**	
	Data year	T (N=7)	C (N=51)	T (N=21)	C (N=81)	T (N=25)	C (N=58)	T (N=29)	C (N=59)	T (N=6)	C (N=248)	T (N=102)	C (N=31)

Monthly Medicaid enrollment rate, sample beneficiaries age <65	3 years prior to MMP roll-out	60.6	60.8	59.4	49.8	50.7	49.5	39.1	31.6	28.4	28.7	30.5	23.0
Monthly Medicaid enrollment rate, sample beneficiaries age ≥65		39.4	34.8	24.5	11.8	19.7	14.7	14.1	10.0	18.7	16.0	12.0	11.0
Continuous annual Medicaid enrollment rate, sample beneficiaries age <65	3 years prior to MMP roll-out	55.6	55.9	57.0	48.4	45.8	47.0	31.2	26.0	25.3	25.4	28.4	23.0
Continuous annual Medicaid enrollment rate, sample beneficiaries age ≥65		35.6	31.5	23.2	10.8	18.4	14.8	11.7	9.3	17.7	14.9	11.3	11.8
Percent MA penetration (mean)	2015	48.8	17.0	25.3	16.4	30.4	29.8	43.5	31.6	42.1	21.7	16.9	20.4
Percent in poverty (mean)	2015	13.6	16.7	12.9	13.9	15.8	15.4	12.4	14.9	19.1	16.8	13.8	17.8
Percent White (mean)	2010	38.7	59.6	80.6	90.5	85.7	90.7	85.8	93.9	28.2	60.0	69.5	84.4
Percent Black (mean)	2010	4.8	2.8	7.8	4.1	5.6	3.0	7.9	2.1	10.8	6.0	21.2	10.4
Percent Hispanic (mean)	2010	37.2	27.3	7.2	3.6	2.5	3.2	2.9	1.9	56.2	31.7	4.9	3.1
Rural county (%)	2013	0.0	82.4	76.2	85.2	92.0	93.1	51.7	89.8	33.3	87.5	56.3	90.3
N short term gen hospital beds per 1000 residents (mean)	2015	1.71	1.77	2.44	1.81	2.71	2.03	2.06	1.61	2.52	1.93	2.10	4.50
N nursing home beds per 1000 residents (mean)	2015	0.08	0.40	0.18	0.27	0.80	0.34	0.08	0.04	0.11	0.14	0.08	1.07
Physicians per 1000 residents (mean)	2015	2.95	1.83	2.05	0.78	1.26	1.29	2.21	0.75	2.37	0.73	1.94	1.38

Notes: “T” designates treatment counties and “C” designates comparison counties. Treatment counties are defined as counties offering MMPs in Financial Alignment Demonstration states. Neighbor-state comparison counties are counties in neighboring states without MMPs. Within-state comparison counties are counties not offering MMPs in Financial Alignment Demonstration states.
Source: Area Health Resources Files 2020

Appendix Table 2: Differential change in Medicare Advantage penetration rates between MMP counties and two regional comparison groups
MA penetration rates, %

Treatment group 1		Comparison group 1		Differential change	Treatment group 2		Comparison group 2		Differential change
**2013**	**2018**	**2013**	**2018**		**2013**	**2018**	**2013**	**2018**	
20.7	25.7	19.3	24.7	-0.4	20.6	25.7	18.8	25.8	-1.9

Notes: Comparison group 1 is comprised of counties in non-MMP states bordering the MMP state. Comparison group 2 is comprised of counties without MMP availability within the MMP state.

Source: Area Health Resources Files 2020

Appendix Figure 2: MMP enrollment rates in demonstration counties of nine analyzed Financial Alignment Initiative states

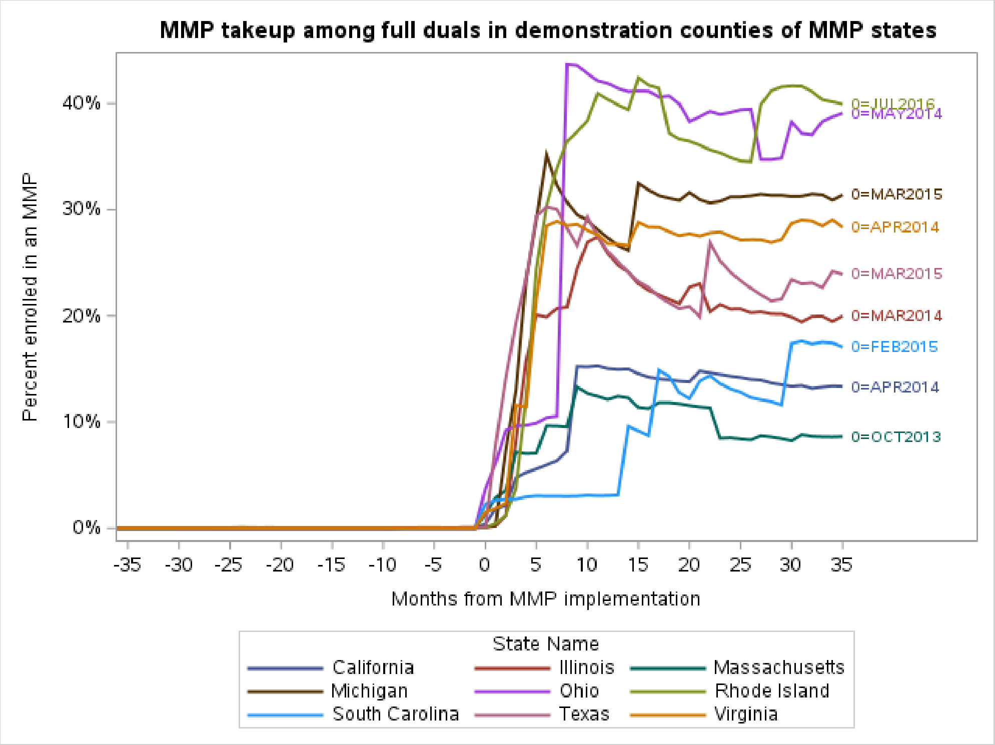

Notes: Month 0 is the first month of MMP enrollment in the state. The calendar month and year of MMP enrollment are indicated to the right of the graph. In Massachusetts and South Carolina, the denominator is limited to full dual-eligible individuals aged <65 and $\geq$65, respectively, reflecting the MMP eligibility requirements in those states. South Carolina excluded dual-eligible beneficiaries enrolled in Medicaid 1915(c) HCBS waiver programs from the state's MMP. Because we do not observe HCBS waiver enrollment, the denominator of MMP-eligible individuals reported is smaller, and the rate of MMP participation among eligible individuals is higher, in the federal evaluations of South Carolina's program. The number of MMP enrollees in Ohio we identified closely matches those of federally funded evaluations but the number of eligible dual-eligible individuals eligible for MMP enrollment (denominator of our rate) is larger in our sample than reported in federally funded evaluations. Consequently, this plot shows a lower rate of MMP take-up than federally funded evaluations. However, the time trend of MMP enrollment is similar to what has been reported previously.

Source: Master Beneficiary Summary File 20% sample 2010-2019, CMS Financial Alignment Initiative reports

Appendix Figure 3: Map of MMP treatment counties and neighbor-state and within-state comparison counties

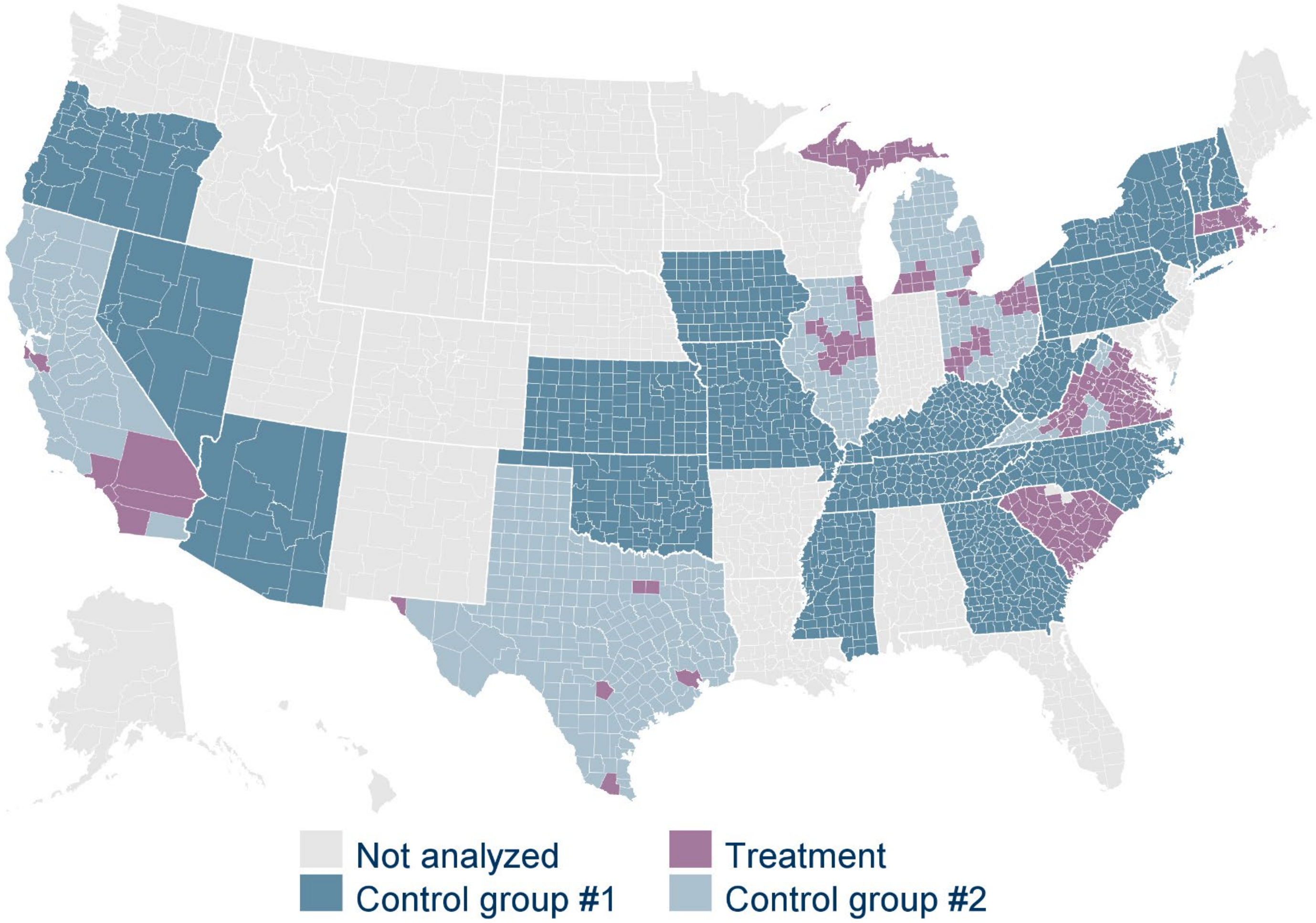

Notes: "Treatment" counties are counties offering MMPs in Financial Alignment Demonstration states. "Comparison group #1" counties are counties in neighboring states without MMPs. Comparison group #1 counties are matched with treatment counties on Medicaid expansion status (see methods for details). "Comparison group #2" counties are counties not offering MMPs within Financial Alignment Demonstration states.

Appendix Figure 4a: Individual event-study estimates of monthly Medicaid enrollment, among residents of 20% highest-poverty zip codes

Comparison group #1 (non-MMP counties in neighboring states)

Monthly medicaid enrollment, among Medicare beneficiaries aged <65 | Monthly medicaid enrollment, among Medicare beneficiaries aged >=65

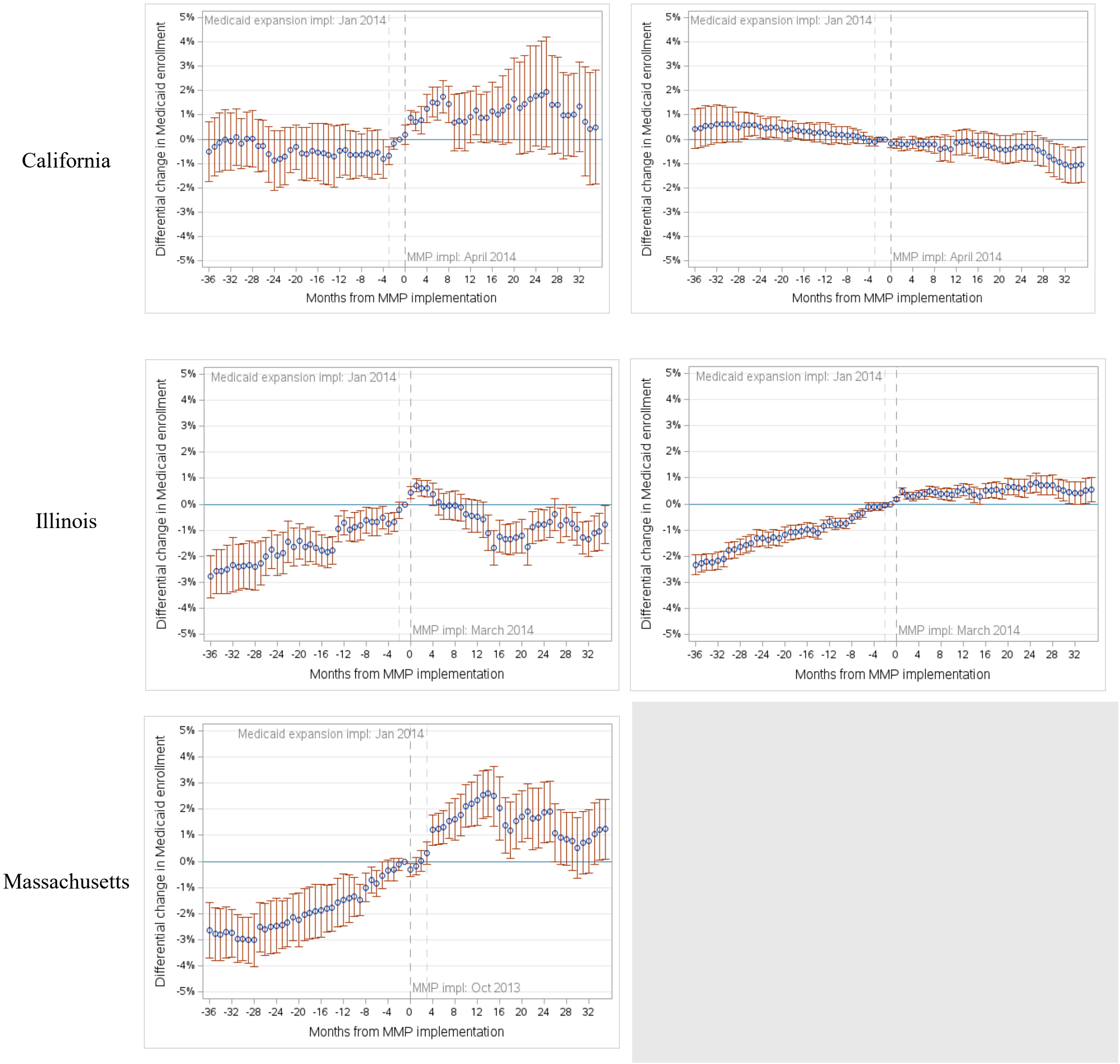

Note: Some Y-axes are differently scaled to accommodate varied differential ranges. Estimates are among Medicare beneficiaries living in the 20% of zip codes with the highest poverty rates among individuals ages 65 and older. Figures depict event-study estimates of the differential change in enrollment in full Medicaid between the treatment group (residents of counties with MMPs in Financial Alignment Demonstration states) vs. comparison groups. The outcome is a binary person-month-level indicator for enrollment in full Medicaid in months when a beneficiary was alive and enrolled in Medicare. Comparison group #1 includes Medicare beneficiaries living in non-MMP states neighboring the Financial Alignment Demonstration state, matched on ACA Medicaid expansion status (see Table 1). Comparison group #2 includes Medicare beneficiaries living in counties of the state where MMPs were not implemented (limited to 6 states where MMPs were implemented in a subset of counties). Event-study estimates obtained from a beneficiary-month-level linear regression model predicting Medicaid enrollment as a function of treatment state indicators, event-time, and the interaction of these terms. Estimates adjusted for sex, race and ethnicity, and original reason for Medicare eligibility. 95% confidence intervals constructed using standard errors clustered by county.

Source: Master Beneficiary Summary File 2010-2019 20% sample, CMS Financial Alignment Initiative reports, American Community Survey 2019

Appendix Figure 4b: Individual event-study estimates of monthly Medicaid enrollment, among residents of 20% highest-poverty zip codes

Comparison group #1 (non-MMP counties in neighboring states)

	Monthly medicaid enrollment, among Medicare beneficiaries aged <65	Monthly medicaid enrollment, among Medicare beneficiaries aged >=65

Michigan

Ohio

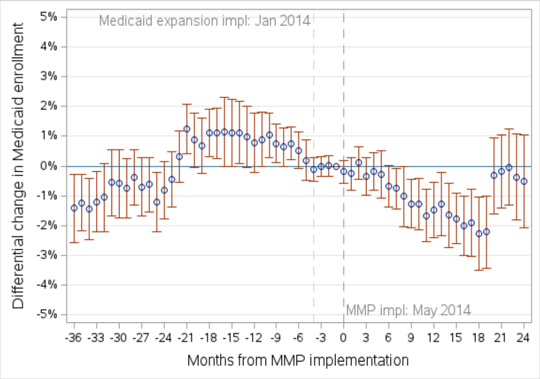

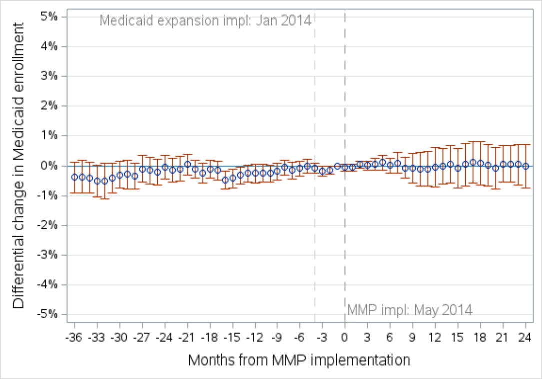

Rhode Island

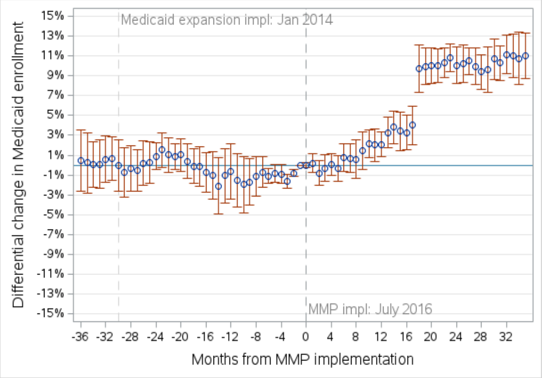

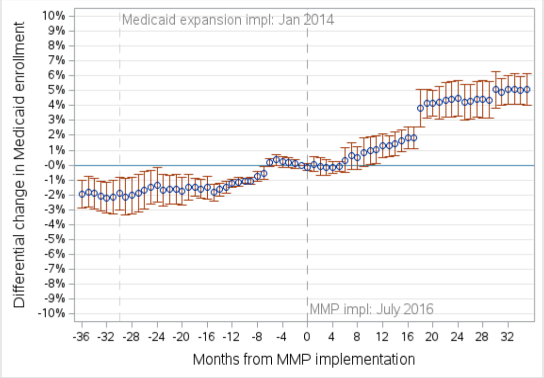

Note: Some Y-axes are differently scaled to accommodate varied differential ranges. Estimates are among Medicare beneficiaries living in the 20% of zip codes with the highest poverty rates among individuals ages 65 and older. Figures depict event-study estimates of the differential change in enrollment in full Medicaid between the treatment group (residents of counties with MMPs in Financial Alignment Demonstration states) vs. comparison groups. The outcome is a binary person-month-level indicator for enrollment in full Medicaid in months when a beneficiary was alive and enrolled in Medicare. Comparison group #1 includes Medicare beneficiaries living in non-MMP states neighboring the Financial Alignment Demonstration state, matched on ACA Medicaid expansion status (see Table 1). Comparison group #2 includes Medicare beneficiaries living in counties of the state where MMPs were not implemented (limited to 6 states where MMPs were implemented in a subset of counties). Event-study estimates obtained from a beneficiary-month-level linear regression model predicting Medicaid enrollment as a function of treatment state indicators, event-time, and the interaction of these terms. Estimates adjusted for sex, race and ethnicity, and original reason for Medicare eligibility. 95% confidence intervals constructed using standard errors clustered by county.

Source: Master Beneficiary Summary File 2010-2019 20% sample, CMS Financial Alignment Initiative reports, American Community Survey 2019

Appendix Figure 4c: Individual event-study estimates of monthly Medicaid enrollment, among residents of 20% highest-poverty zip codes

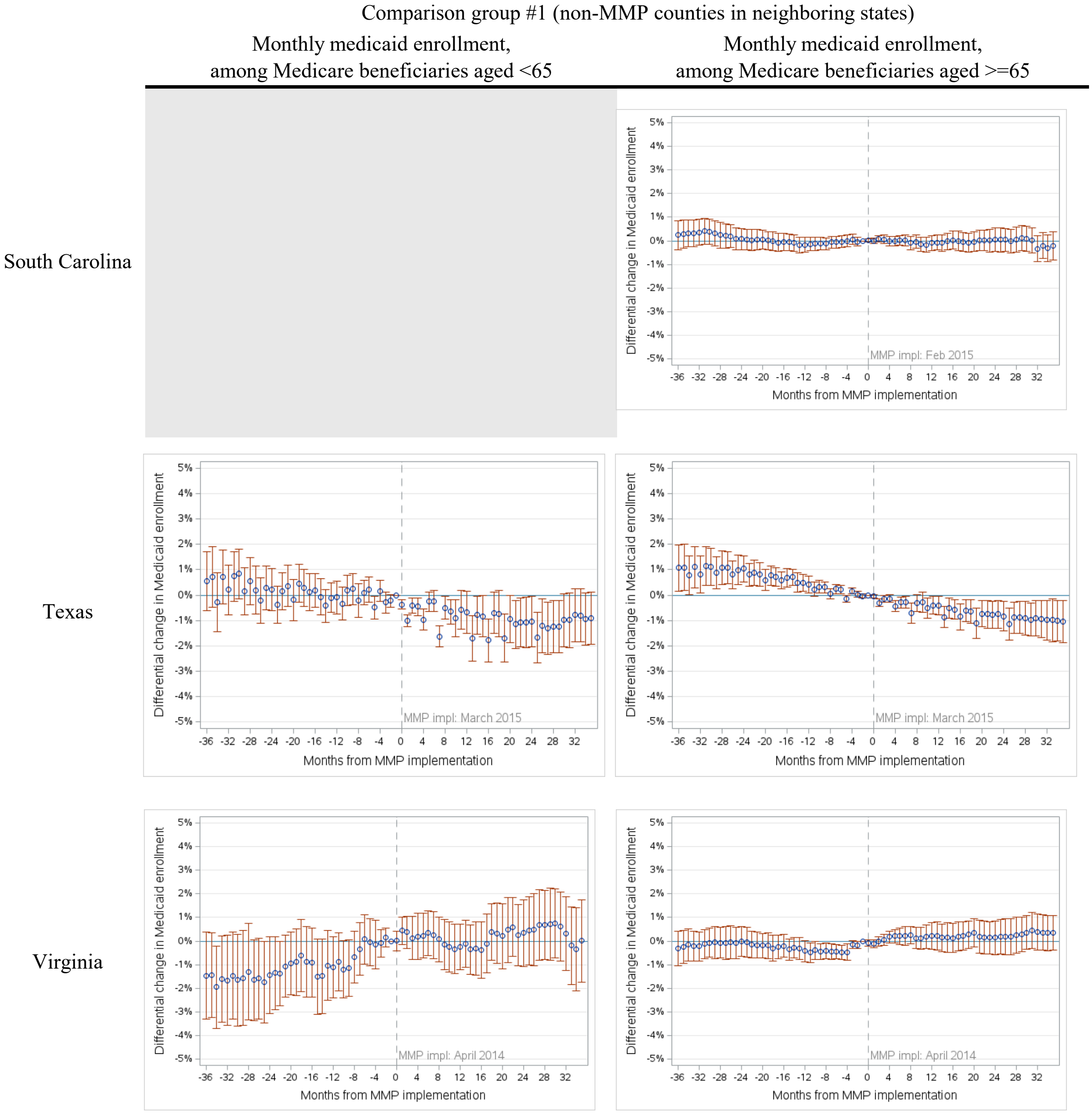

Note: Some Y-axes are differently scaled to accommodate varied differential ranges. Estimates are among Medicare beneficiaries living in the 20% of zip codes with the highest poverty rates among individuals ages 65 and older. Figures depict event-study estimates of the differential change in enrollment in full Medicaid between the treatment group (residents of counties with MMPs in Financial Alignment Demonstration states) vs. comparison groups. The outcome is a binary person-month-level indicator for enrollment in full Medicaid in months when a beneficiary was alive and enrolled in Medicare. Comparison group #1 includes Medicare beneficiaries living in non-MMP states neighboring the Financial Alignment Demonstration state, matched on ACA Medicaid expansion status (see Table 1). Comparison group #2 includes Medicare beneficiaries living in counties of the state where MMPs were not implemented (limited to 6 states where MMPs were implemented in a subset of counties). Event-study estimates obtained from a beneficiary-month-level linear regression model predicting Medicaid enrollment as a function of treatment state indicators, event-time, and the interaction of these terms. Estimates adjusted for sex, race and ethnicity, and original reason for Medicare eligibility. 95% confidence intervals constructed using standard errors clustered by county.

Source: Master Beneficiary Summary File 2010-2019 20% sample, CMS Financial Alignment Initiative reports, American Community Survey 2019

Appendix Figure 4d: Individual event-study estimates of monthly Medicaid enrollment, among residents of 20% highest-poverty zip codes

Comparison group #2 (non-MMP counties in the Financial Alignment Demonstration state)

	Monthly medicaid enrollment, among Medicare beneficiaries aged <65	Monthly medicaid enrollment, among Medicare beneficiaries aged >=65
California	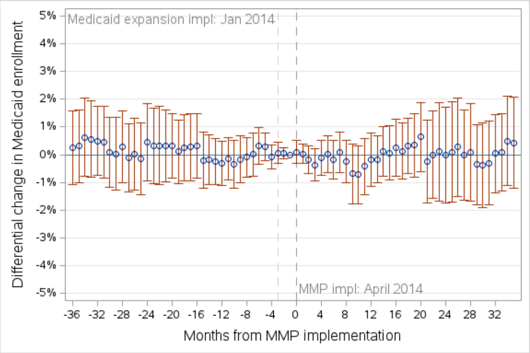	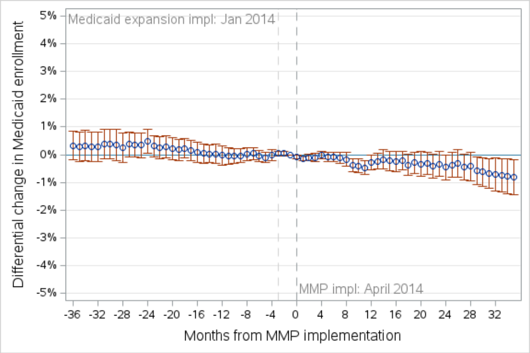
Illinois	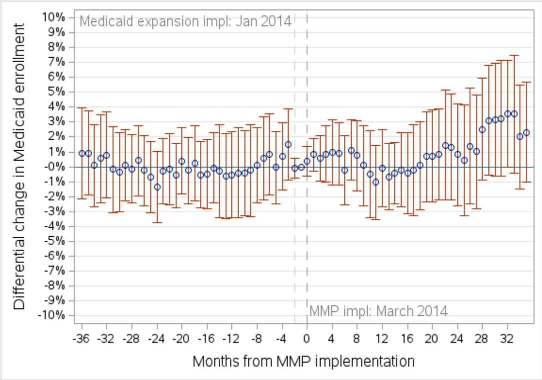	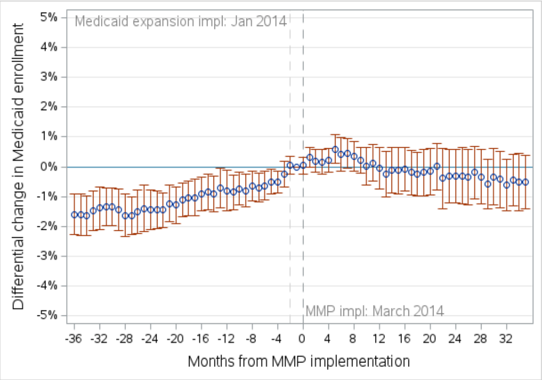
Massachusetts		

Note: Some Y-axes are differently scaled to accommodate varied differential ranges. Estimates are among Medicare beneficiaries living in the 20% of zip codes with the highest poverty rates among individuals ages 65 and older. Figures depict event-study estimates of the differential change in enrollment in full Medicaid between the treatment group (residents of counties with MMPs in Financial Alignment Demonstration states) vs. comparison groups. The outcome is a binary person-month-level indicator for enrollment in full Medicaid in months when a beneficiary was alive and enrolled in Medicare. Comparison group #1 includes Medicare beneficiaries living in non-MMP states neighboring the Financial Alignment Demonstration state, matched on ACA Medicaid expansion status (see Table 1). Comparison group #2 includes Medicare beneficiaries living in counties of the state where MMPs were not implemented (limited to 6 states where MMPs were implemented in a subset of counties). Event-study estimates obtained from a beneficiary-month-level linear regression model predicting Medicaid enrollment as a function of treatment state indicators, event-time, and the interaction of these terms. Estimates adjusted for sex, race and ethnicity, and original reason for Medicare eligibility. 95% confidence intervals constructed using standard errors clustered by county.

Source: Master Beneficiary Summary File 2010-2019 20% sample, CMS Financial Alignment Initiative reports, American Community Survey 2019

Appendix Figure 4e: Individual event-study estimates of monthly Medicaid enrollment, among residents of 20% highest-poverty zip codes

Comparison group #2 (non-MMP counties in the Financial Alignment Demonstration state)

	Monthly medicaid enrollment, among Medicare beneficiaries aged <65	Monthly medicaid enrollment, among Medicare beneficiaries aged >=65
Michigan	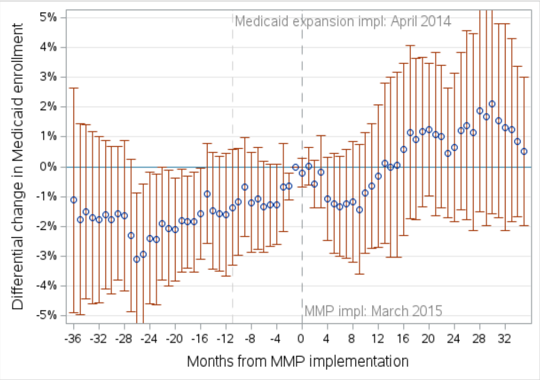	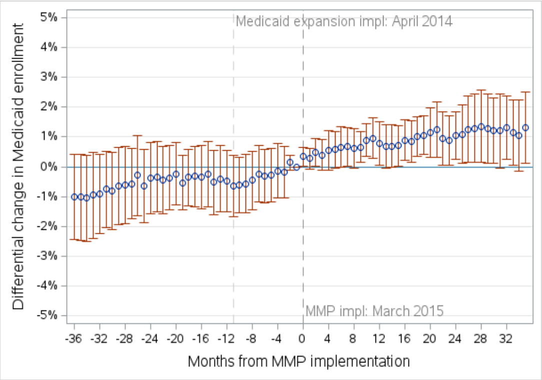
Ohio	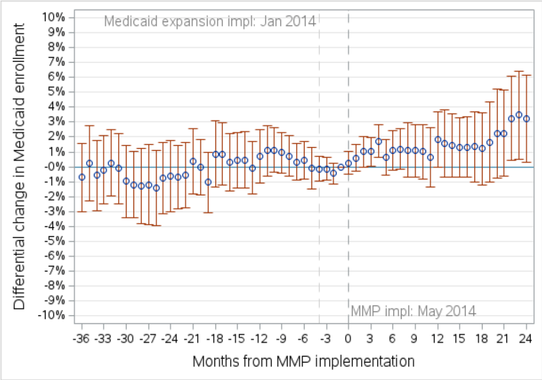	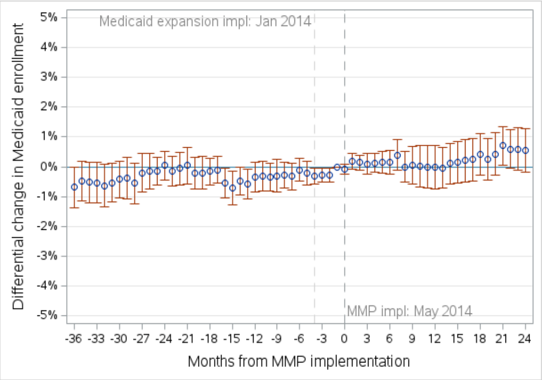
Rhode Island		

Note: Some Y-axes are differently scaled to accommodate varied differential ranges. Estimates are among Medicare beneficiaries living in the 20% of zip codes with the highest poverty rates among individuals ages 65 and older. Figures depict event-study estimates of the differential change in enrollment in full Medicaid between the treatment group (residents of counties with MMPs in Financial Alignment Demonstration states) vs. comparison groups. The outcome is a binary person-month-level indicator for enrollment in full Medicaid in months when a beneficiary was alive and enrolled in Medicare. Comparison group #1 includes Medicare beneficiaries living in non-MMP states neighboring the Financial Alignment Demonstration state, matched on ACA Medicaid expansion status (see Table 1). Comparison group #2 includes Medicare beneficiaries living in counties of the state where MMPs were not implemented (limited to 6 states where MMPs were implemented in a subset of counties). Event-study estimates obtained from a beneficiary-month-level linear regression model predicting Medicaid enrollment as a function of treatment state indicators, event-time, and the interaction of these terms. Estimates adjusted for sex, race and ethnicity, and original reason for Medicare eligibility. 95% confidence intervals constructed using standard errors clustered by county.
Source: Master Beneficiary Summary File 2010-2019 20% sample, CMS Financial Alignment Initiative reports, American Community Survey 2019

Appendix Figure 4f: Individual event-study estimates of monthly Medicaid enrollment, among residents of 20% highest-poverty zip codes

Comparison group #2 (non-MMP counties in the Financial Alignment Demonstration state)

	Monthly medicaid enrollment, among Medicare beneficiaries aged <65	Monthly medicaid enrollment, among Medicare beneficiaries aged >=65
South Carolina		

Texas

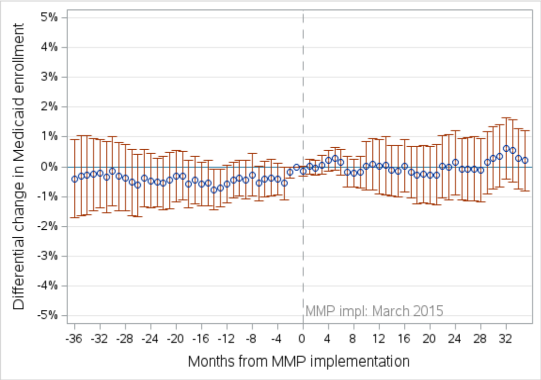

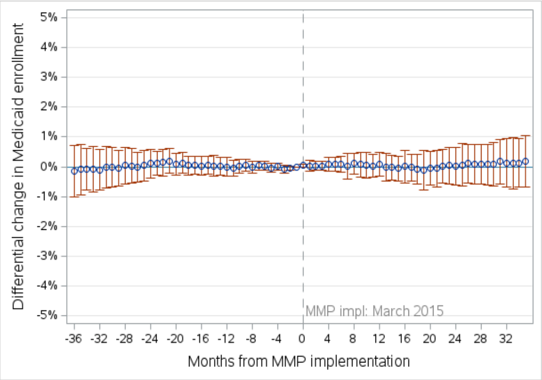

Virginia

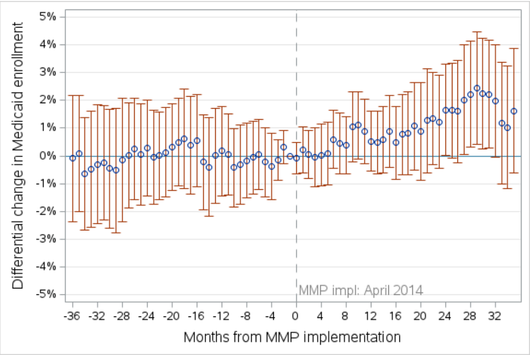

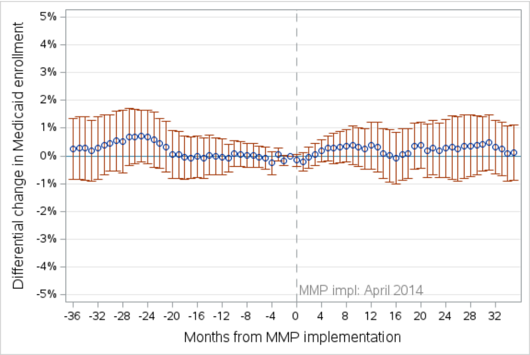

Note: Some Y-axes are differently scaled to accommodate varied differential ranges. Estimates are among Medicare beneficiaries living in the 20% of zip codes with the highest poverty rates among individuals ages 65 and older. Figures depict event-study estimates of the differential change in enrollment in full Medicaid between the treatment group (residents of counties with MMPs in Financial Alignment Demonstration states) vs. comparison groups. The outcome is a binary person-month-level indicator for enrollment in full Medicaid in months when a beneficiary was alive and enrolled in Medicare. Comparison group #1 includes Medicare beneficiaries living in non-MMP states neighboring the Financial Alignment Demonstration state, matched on ACA Medicaid expansion status (see Table 1). Comparison group #2 includes Medicare beneficiaries living in counties of the state where MMPs were not implemented (limited to 6 states where MMPs were implemented in a subset of counties). Event-study estimates obtained from a beneficiary-month-level linear regression model predicting Medicaid enrollment as a function of treatment state indicators, event-time, and the interaction of these terms. Estimates adjusted for sex, race and ethnicity, and original reason for Medicare eligibility. 95% confidence intervals constructed using standard errors clustered by county.

Source: Master Beneficiary Summary File 2010-2019 20% sample, CMS Financial Alignment Initiative reports, American Community Survey 2019

Appendix Table 3: Difference-in-differences estimates of continuous Medicaid enrollment, among residents of 20% highest-poverty zip codes

Panel A. Continuous annual Medicaid enrollment, among beneficiaries < 65 years

	**Treatment group vs. comparison group #1** (a)				**Treatment group vs. comparison group #2** (b)			
	Differential trend pre-MMP implementation (c)		**DID estimate,** post vs. pre-MMP implementation		Differential trend pre-MMP implementation (c)		**DID estimate,** post vs. pre-MMP implementation	
	Estimate, percentage points (pp)	*95% CI*	*Estimate, pp*	*95% CI*	*Estimate, pp*	*95% CI*	*Estimate, pp*	*95% CI*
Pooled DID estimate (d)	0.39	-0.14, 0.92	0.64	-0.23, 1.50	-0.01	-0.77, 0.75	0.32	-0.60, 1.24
State-specific estimates: (e)								
California	-0.54	-1.44, 0.36	1.25	-0.61, 3.12	-0.88	-1.98, 0.22	-0.45	-2.33, 1.42
Illinois	0.49	-0.17, 1.14	-1.57	-2.73, -0.42	-1.10	-4.10, 1.90	-2.39	-6.07, 1.29
Massachusetts	0.93	0.16, 1.70	3.92	2.55, 5.29				
Michigan					1.82	-0.18, 3.82	3.79	0.80, 6.77
Ohio	1.95	1.19, 2.70	0.94	-0.27, 2.15	2.55	0.32, 4.77	4.35	2.19, 6.52
Rhode Island	-1.47	-3.26, 0.31	8.12	3.31, 12.92				
South Carolina								
Texas	0.31	-0.39, 1.02	-1.24	-2.62, 0.14	0.12	-0.68, 0.92	0.06	-1.42, 1.53
Virginia	-0.18	-2.15, 1.79	-1.18	-4.10, 1.75	0.09	-1.87, 2.05	-1.20	-4.60, 2.20

Panel B. Continuous annual Medicaid enrollment, among beneficiaries ≥ 65 years

	**Treatment group vs. comparison group #1** (a)				**Treatment group vs. comparison group #2** (b)			
	Differential trend pre-MMP implementation (c)		**DID estimate,** post vs. pre-MMP implementation		Differential trend pre-MMP implementation (c)		**DID estimate,** post vs. pre-MMP implementation	
	Estimate, pp	*95% CI*	*Estimate, pp*	*95% CI*	*Estimate, pp*	*95% CI*	*Estimate, pp*	*95% CI*
Pooled DID estimate (d)	-0.43	-1.22, 0.36	-0.58	-1.33, 0.17	-0.15	-0.77, 0.48	-0.33	-1.01, 0.35
State-specific estimates: (e)								
California	-0.15	-0.51, 0.21	-0.77	-1.60, 0.05	-0.22	-0.56, 0.13	-1.01	-1.68, -0.33
Illinois	0.39	0.12, 0.66	1.07	0.53, 1.60	-0.22	-0.94, 0.51	0.70	-0.56, 1.95
Massachusetts								
Michigan					0.55	-0.27, 1.37	1.95	0.89, 3.01
Ohio	0.34	0.01, 0.67	0.84	0.01, 1.68	0.06	-0.50, 0.62	1.56	0.45, 2.68
Rhode Island	1.12	0.36, 1.89	6.34	4.54, 8.14				

South Carolina	0.15	-0.31, 0.61	0.19	-0.80, 1.19				
Texas	-0.29	-0.74, 0.16	-1.50	-2.32, -0.69	-0.03	-0.43, 0.38	-0.12	-0.98, 0.75
Virginia	-0.45	-1.02, 0.12	-0.03	-0.82, 0.77	-0.26	-1.15, 0.63	0.12	-1.08, 1.32

Notes: Difference-in-differences (DID) estimates reflect differential change in continuous enrollment in full Medicaid between the treatment groups (residents of counties with MMPs in Financial Alignment Demonstration States) vs. comparison groups for these states (see methods for details). The outcome is a binary person-year-level indicator for continuous enrollment in full Medicaid in months when a beneficiary was alive and enrolled in Medicare. Estimates are in percentage points (pp). Estimates were obtained from a beneficiary-year-level linear regression model predicting continuous Medicaid enrollment as a function of treatment state indicators, event-time, and the interaction of these terms. Estimates are adjusted for sex, race and ethnicity, and original reason for Medicare eligibility. 95% confidence intervals constructed using standard errors clustered by county. Shaded cells denote where analyses were not conducted because the MMP was not implemented in the age group shown (e.g., Massachusetts' MMP for beneficiaries ages >65 years) or because a comparison group was not available.
(a) Comparison group #1 includes Medicare beneficiaries living in non-MMP states neighboring states that implemented MMPs, matched on ACA Medicaid expansion status (see methods for details).
(b) Comparison group #2 includes Medicare beneficiaries living in non-MMP counties in Financial Alignment Demonstration states. This analysis excludes Massachusetts, South Carolina, and Rhode Island, whose MMP programs are statewide or nearly statewide.
(c) Differential pre-MMP implementation trend reported as the annual average differential change in enrollment in full Medicaid between treatment and comparison groups during the 3-year period prior to MMP implementation.
(d) Pooled difference-in-differences estimates aggregate over the state-specific estimates using the method of stacked difference-in-differences.
(e) State-specific estimates obtained from separate models fitted on observations from each state and comparison group. See Appendix Figure 6 for event-study plots depicting differential changes in continuous Medicaid enrollment in each treatment state vs. control states.
Sources: Master Beneficiary Summary File 2010-2019 enhanced 5% sample, American Community Survey 2019, CMS Financial Alignment Initiative reports.

Appendix Figure 5: Event-study estimates of continuous annual Medicaid enrollment while alive, among residents of 20% highest-poverty zip codes

Panel A: Beneficiaries under age 65, treatment group vs. comparison group #1

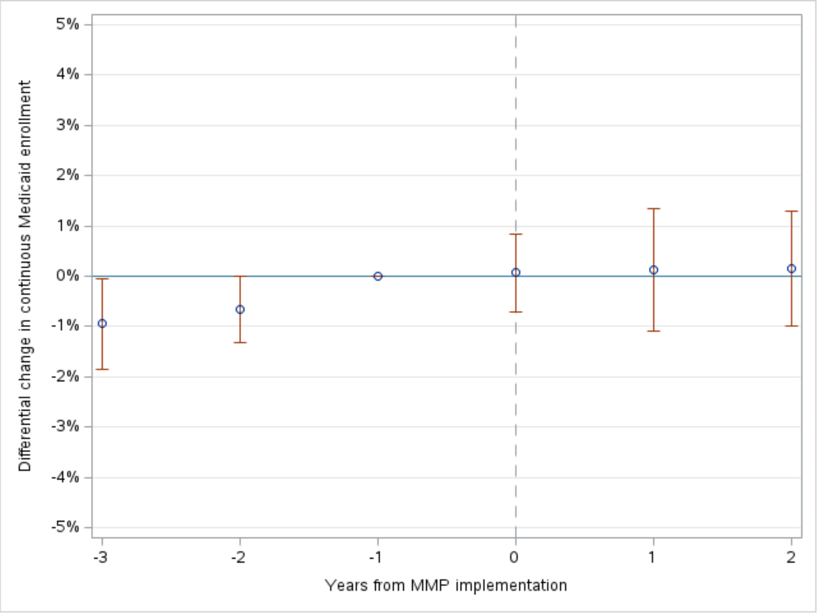

Panel B: Beneficiaries under age 65, treatment group vs. comparison group #2

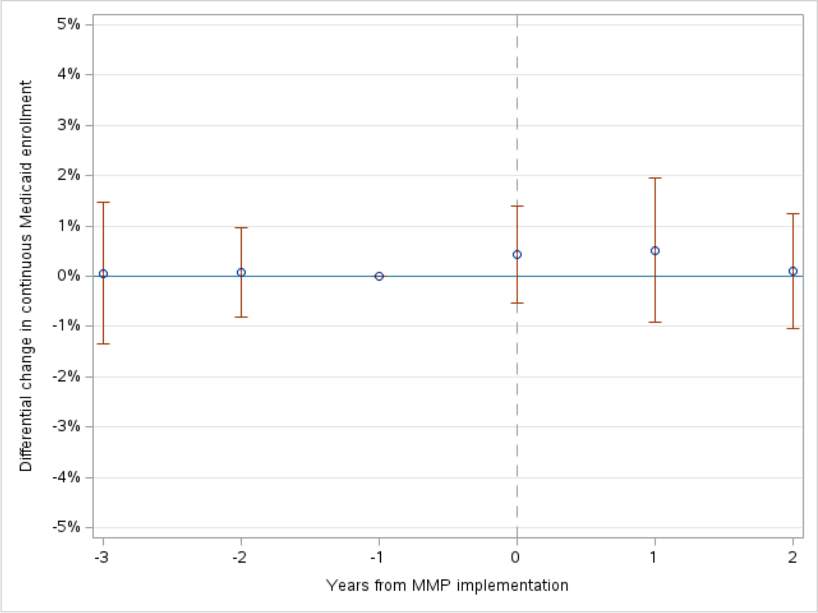

**Panel C:** Beneficiaries aged 65 and older, treatment group vs. comparison group #1	**Panel D:** Beneficiaries aged 65 and older, treatment group vs. comparison group #2

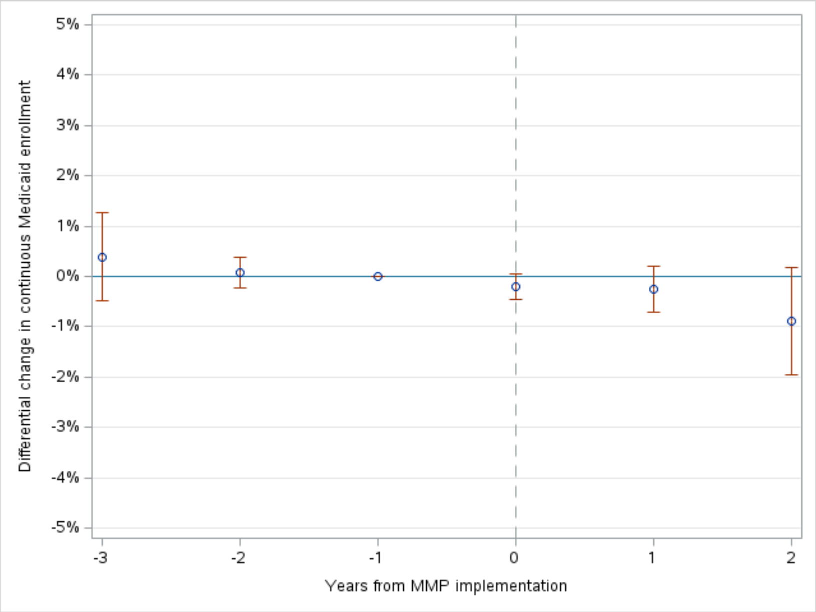

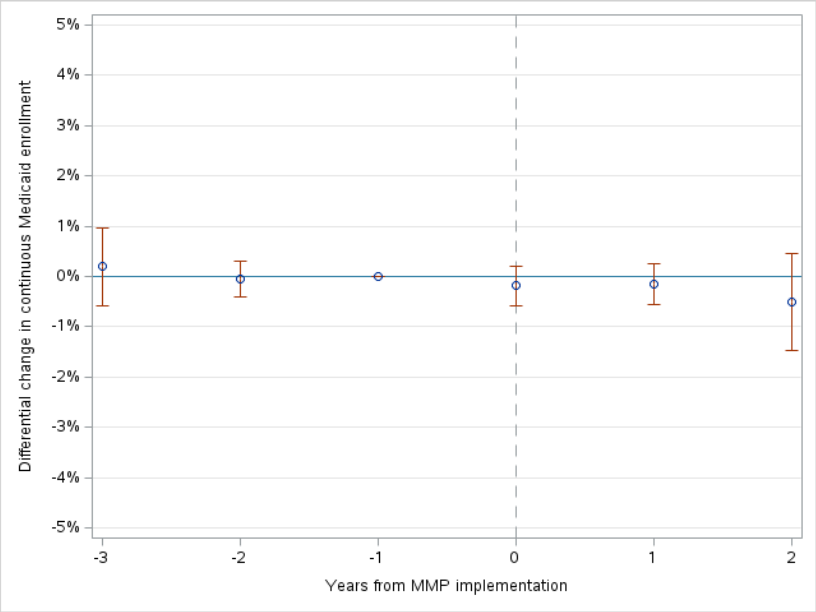

Notes: Estimates are among Medicare beneficiaries living in the 20% of zip codes with the highest poverty rates among individuals ages 65 and older. Figures depict event-study estimates of the differential change in continuous annual enrollment in full Medicaid (while alive) between the treatment group (residents of counties with MMPs in Financial Alignment Demonstration states) vs. comparison groups. The outcome is a binary person-year-level indicator for enrollment in full Medicaid in all months while a beneficiary was alive and enrolled in Medicare. Comparison group #1 includes Medicare beneficiaries living in non-MMP states neighboring those that implemented MMPs, matched on ACA Medicaid expansion status (see Table 1). Comparison group #2 includes Medicare beneficiaries living in counties of states where MMPs were not implemented (limited to 6 states where MMPs were implemented in a subset of counties). Event-study estimates obtained from a beneficiary-year-level linear regression model predicting continuous Medicaid enrollment as a function of treatment state indicators, event-time, and the interaction of these terms. Estimates adjusted for sex, race and ethnicity, and original reason for Medicare eligibility. 95% confidence intervals constructed using standard errors clustered by county. Panels A-B are among beneficiaries under age 65 in the study period and Panels C-D are among beneficiaries ages 65 and older in the study period.

Sources: Master Beneficiary Summary File 2010-2019 enhanced 5% sample, American Community Survey 2019, CMS Financial Alignment Initiative reports.

Appendix Figure 6a: Individual event-study estimates of continuous annual Medicaid enrollment, among residents of 20% highest-poverty zip codes

Comparison group #1 (non-MMP counties in neighboring states)

	Continuous annual Medicaid enrollment, among Medicare beneficiaries aged <65	Continuous annual Medicaid enrollment, among Medicare beneficiaries aged >=65

California

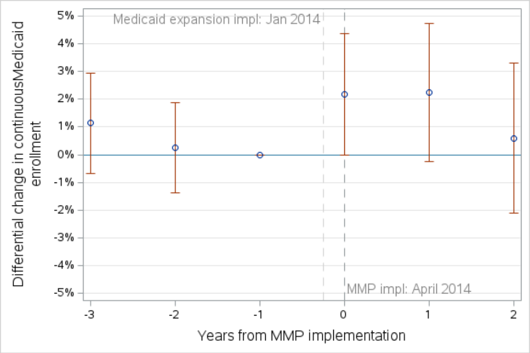

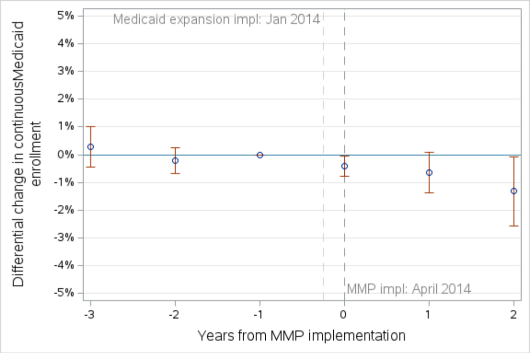

Illinois

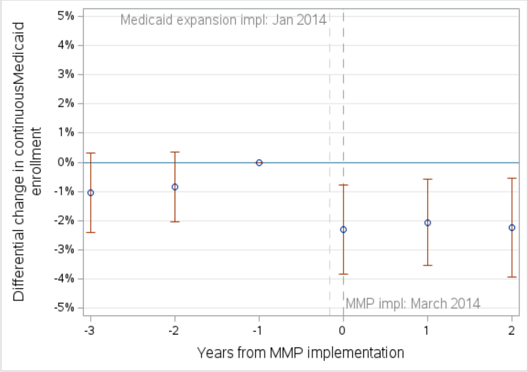

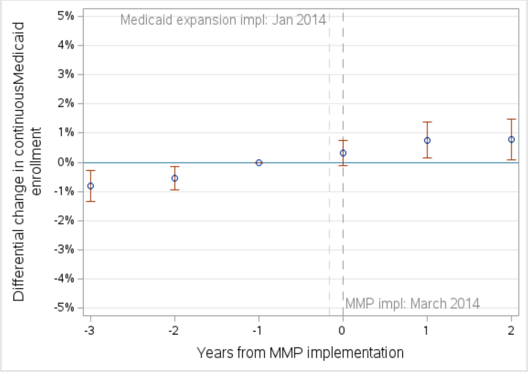

Massachusetts

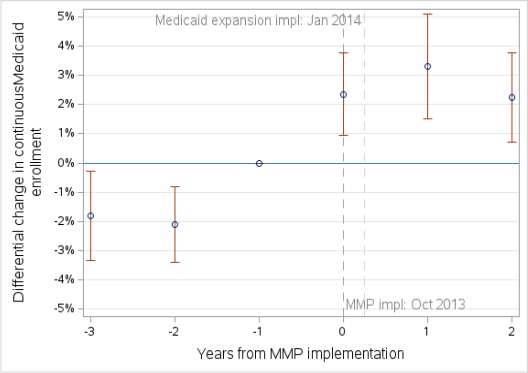

Note: Some Y-axes are differently scaled to accommodate varied differential ranges. Estimates are among Medicare beneficiaries living in the 20% of zip codes with the highest poverty rates among individuals ages 65 and older. Figures depict event-study estimates of the differential change in enrollment in continuous annual full Medicaid between the treatment group (residents of counties with MMPs in Financial Alignment Demonstration states) vs. comparison groups. The outcome is a binary person-year-level indicator for enrollment in full Medicaid in months when a beneficiary was alive and enrolled in Medicare. Comparison group #1 includes Medicare beneficiaries living in non-MMP states neighboring the Financial Alignment Demonstration state, matched on ACA Medicaid expansion status (see Table 1). Comparison group #2 includes Medicare beneficiaries living in counties of the state where MMPs were not implemented (limited to 6 states where MMPs were implemented in a subset of counties). Event-study estimates obtained from a beneficiary-year-level linear regression model predicting Medicaid enrollment as a function of treatment state indicators, event-time, and the interaction of these terms. Estimates adjusted for sex, race and ethnicity, and original reason for Medicare eligibility. 95% confidence intervals constructed using standard errors clustered by county.
Source: Master Beneficiary Summary File 2010-2019 enhanced 5% sample, CMS Financial Alignment Initiative reports, American Community Survey 2019

Appendix Figure 6b: Individual event-study estimates of continuous annual Medicaid enrollment, among residents of 20% highest-poverty zip codes

Comparison group #1 (non-MMP counties in neighboring states)

	Continuous annual Medicaid enrollment, among Medicare beneficiaries aged <65	Continuous annual Medicaid enrollment, among Medicare beneficiaries aged >=65
Michigan		
Ohio	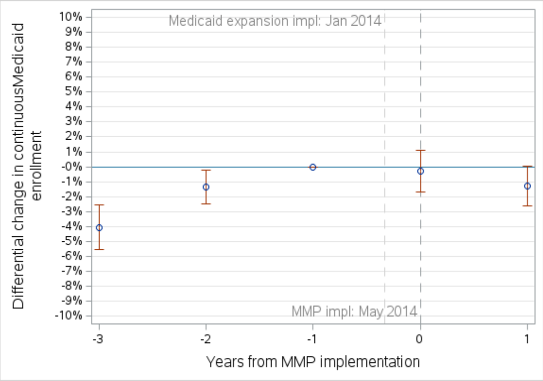	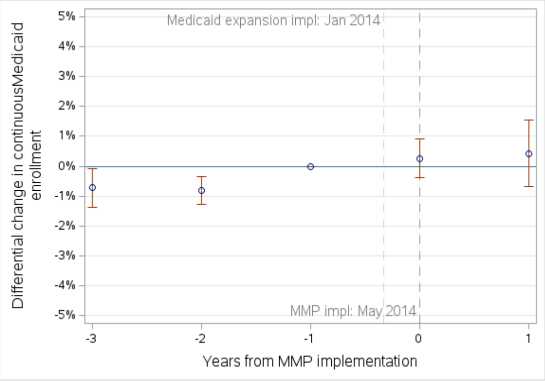
Rhode Island	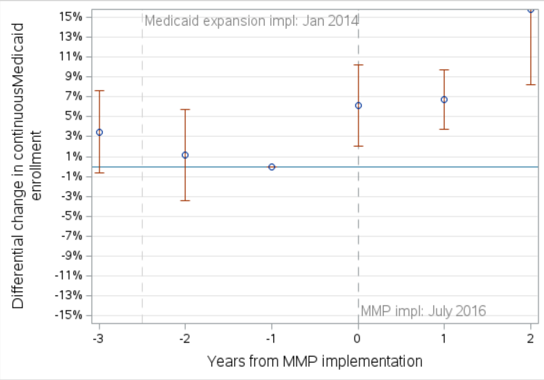	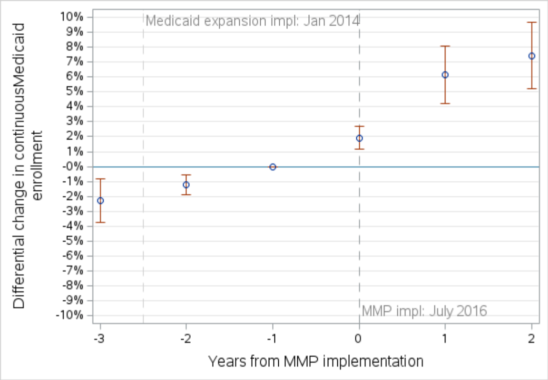

Note: Some Y-axes are differently scaled to accommodate varied differential ranges. Estimates are among Medicare beneficiaries living in the 20% of zip codes with the highest poverty rates among individuals ages 65 and older. Figures depict event-study estimates of the differential change in enrollment in continuous annual full Medicaid between the treatment group (residents of counties with MMPs in Financial Alignment Demonstration states) vs. comparison groups. The outcome is a binary person-year-level indicator for enrollment in full Medicaid in months when a beneficiary was alive and enrolled in Medicare. Comparison group #1 includes Medicare beneficiaries living in non-MMP states neighboring the Financial Alignment Demonstration state, matched on ACA Medicaid expansion status (see Table 1). Comparison group #2 includes Medicare beneficiaries living in counties of the state where MMPs were not implemented (limited to 6 states where MMPs were implemented in a subset of counties). Event-study estimates obtained from a beneficiary-year-level linear regression model predicting Medicaid enrollment as a function of treatment state indicators, event-time, and the interaction of these terms. Estimates adjusted for sex, race and ethnicity, and original reason for Medicare eligibility. 95% confidence intervals constructed using standard errors clustered by county.

Source: Master Beneficiary Summary File 2010-2019 enhanced 5% sample, CMS Financial Alignment Initiative reports, American Community Survey 2019

Appendix Figure 6c: Individual event-study estimates of continuous annual Medicaid enrollment, among residents of 20% highest-poverty zip codes

Comparison group #1 (non-MMP counties in neighboring states)

	Continuous annual Medicaid enrollment, among Medicare beneficiaries aged <65	Continuous annual Medicaid enrollment, among Medicare beneficiaries aged >=65
South Carolina		
Texas		
Virginia		

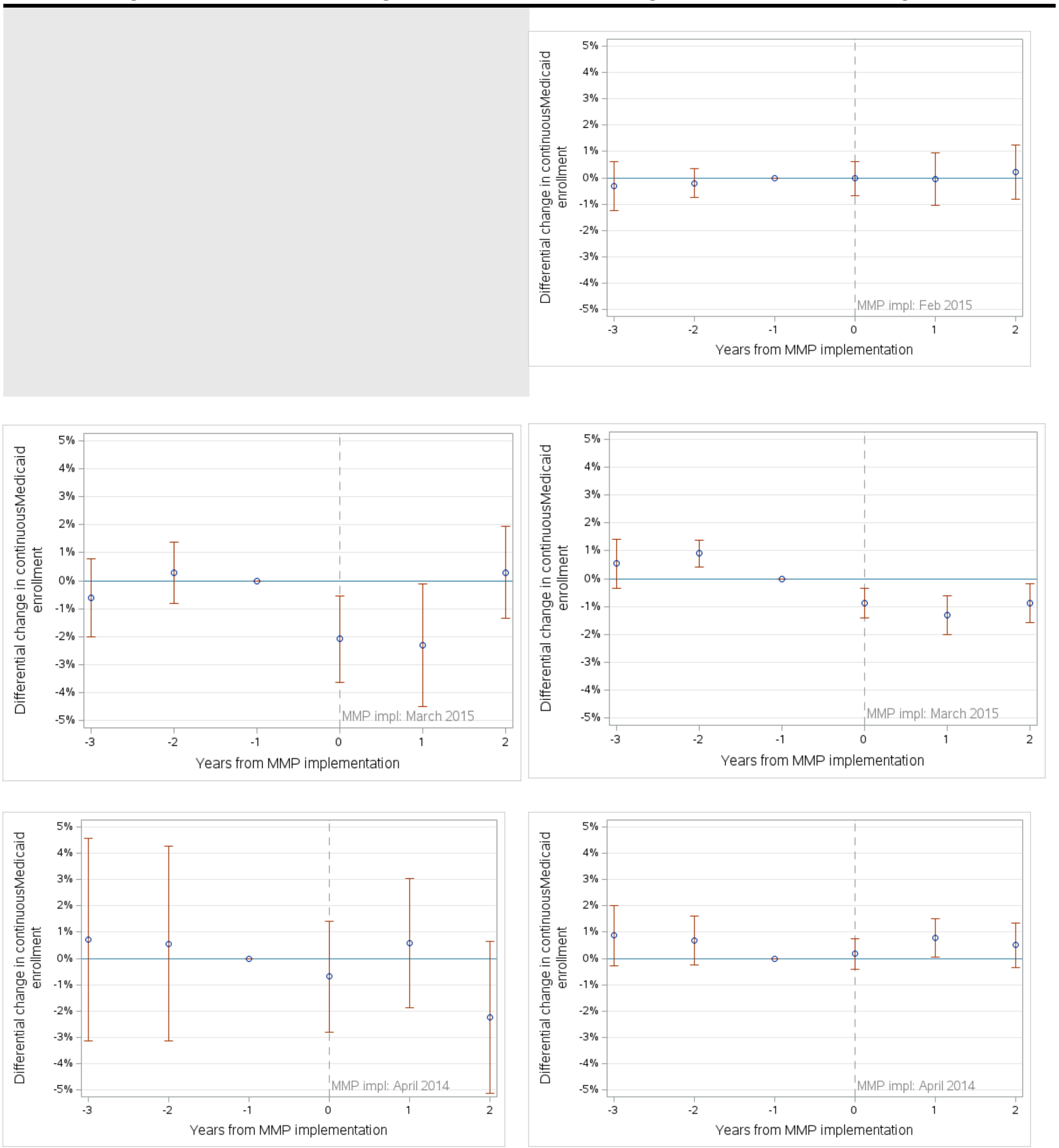

Note: Some Y-axes are differently scaled to accommodate varied differential ranges. Estimates are among Medicare beneficiaries living in the 20% of zip codes with the highest poverty rates among individuals ages 65 and older. Figures depict event-study estimates of the differential change in enrollment in continuous annual full Medicaid between the treatment group (residents of counties with MMPs in Financial Alignment Demonstration states) vs. comparison groups. The outcome is a binary person-year-level indicator for enrollment in full Medicaid in months when a beneficiary was alive and enrolled in Medicare. Comparison group #1 includes Medicare beneficiaries living in non-MMP states neighboring the Financial Alignment Demonstration state, matched on ACA Medicaid expansion status (see Table 1). Comparison group #2 includes Medicare beneficiaries living in counties of the state where MMPs were not implemented (limited to 6 states where MMPs were implemented in a subset of counties). Event-study estimates obtained from a beneficiary-year-level linear regression model predicting Medicaid enrollment as a function of treatment state indicators, event-time, and the interaction of these terms. Estimates adjusted for sex, race and ethnicity, and original reason for Medicare eligibility. 95% confidence intervals constructed using standard errors clustered by county.

Source: Master Beneficiary Summary File 2010-2019 enhanced 5% sample, CMS Financial Alignment Initiative reports, American Community Survey 2019

Appendix Figure 6d: Individual event-study estimates of continuous annual Medicaid enrollment, among residents of 20% highest-poverty zip codes

Comparison group #2 (non-MMP counties in the Financial Alignment Demonstration state)

	Continuous annual Medicaid enrollment, among Medicare beneficiaries aged <65	Continuous annual Medicaid enrollment, among Medicare beneficiaries aged >=65
California	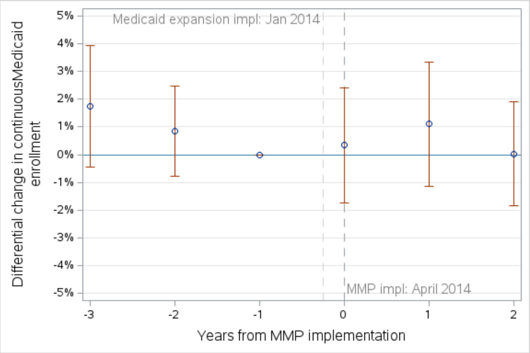	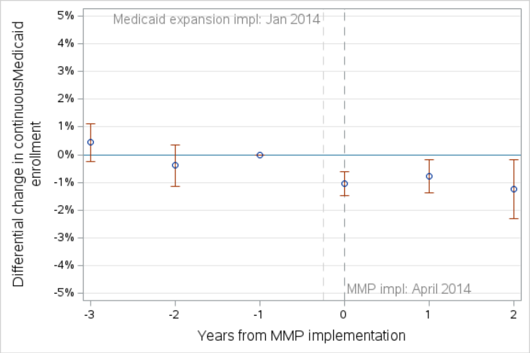
Illinois	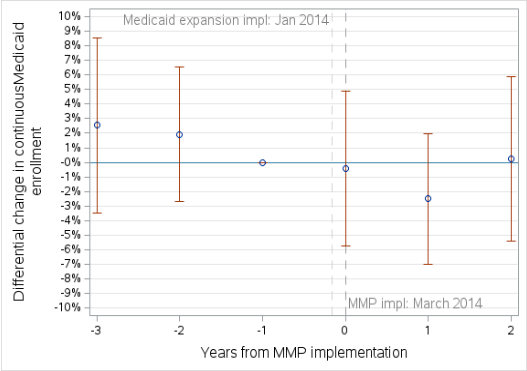	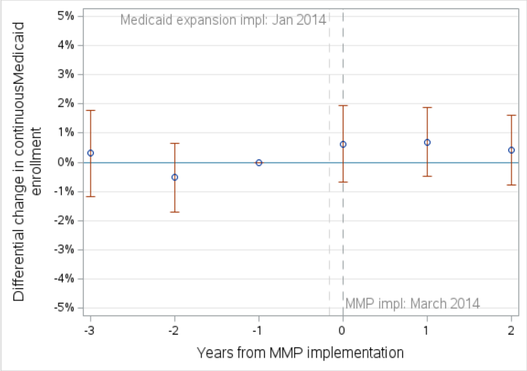
Massachusetts		

Note: Some Y-axes are differently scaled to accommodate varied differential ranges. Estimates are among Medicare beneficiaries living in the 20% of zip codes with the highest poverty rates among individuals ages 65 and older. Figures depict event-study estimates of the differential change in enrollment in continuous annual full Medicaid between the treatment group (residents of counties with MMPs in Financial Alignment Demonstration states) vs. comparison groups. The outcome is a binary person-year-level indicator for enrollment in full Medicaid in months when a beneficiary was alive and enrolled in Medicare. Comparison group #1 includes Medicare beneficiaries living in non-MMP states neighboring the Financial Alignment Demonstration state, matched on ACA Medicaid expansion status (see Table 1). Comparison group #2 includes Medicare beneficiaries living in counties of the state where MMPs were not implemented (limited to 6 states where MMPs were implemented in a subset of counties). Event-study estimates obtained from a beneficiary-year-level linear regression model predicting Medicaid enrollment as a function of treatment state indicators, event-time, and the interaction of these terms. Estimates adjusted for sex, race and ethnicity, and original reason for Medicare eligibility. 95% confidence intervals constructed using standard errors clustered by county.
Source: Master Beneficiary Summary File 2010-2019 enhanced 5% sample, CMS Financial Alignment Initiative reports, American Community Survey 2019

Appendix Figure 6e: Individual event-study estimates of continuous annual Medicaid enrollment, among residents of 20% highest-poverty zip codes

Comparison group #2 (non-MMP counties in the Financial Alignment Demonstration state)

	Continuous annual Medicaid enrollment, among Medicare beneficiaries aged <65	Continuous annual Medicaid enrollment, among Medicare beneficiaries aged >=65
Michigan	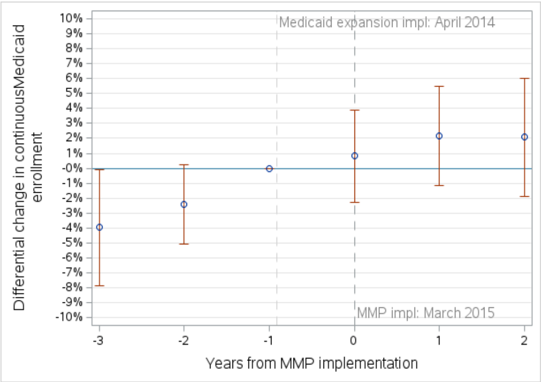	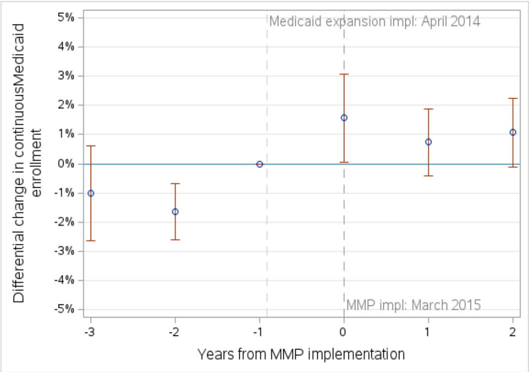
Ohio	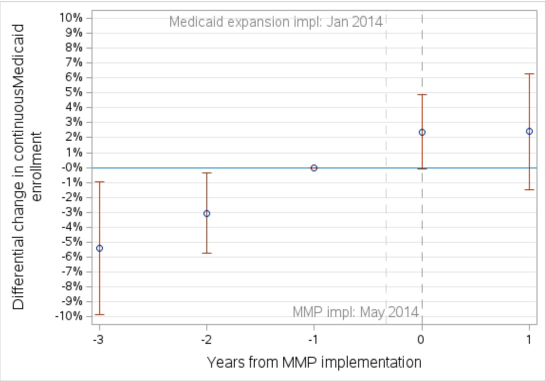	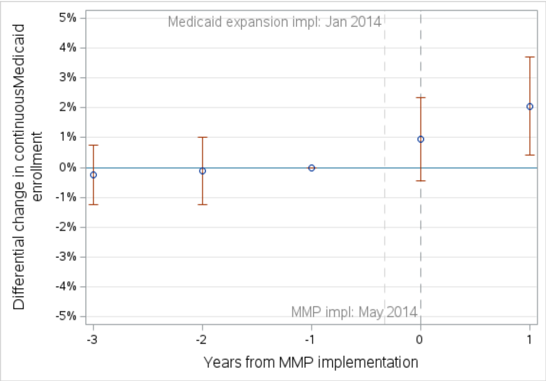
Rhode Island		

Note: Some Y-axes are differently scaled to accommodate varied differential ranges. Estimates are among Medicare beneficiaries living in the 20% of zip codes with the highest poverty rates among individuals ages 65 and older. Figures depict event-study estimates of the differential change in enrollment in continuous annual full Medicaid between the treatment group (residents of counties with MMPs in Financial Alignment Demonstration states) vs. comparison groups. The outcome is a binary person-year-level indicator for enrollment in full Medicaid in months when a beneficiary was alive and enrolled in Medicare. Comparison group #1 includes Medicare beneficiaries living in non-MMP states neighboring the Financial Alignment Demonstration state, matched on ACA Medicaid expansion status (see Table 1). Comparison group #2 includes Medicare beneficiaries living in counties of the state where MMPs were not implemented (limited to 6 states where MMPs were implemented in a subset of counties). Event-study estimates obtained from a beneficiary-year-level linear regression model predicting Medicaid enrollment as a function of treatment state indicators, event-time, and the interaction of these terms. Estimates adjusted for sex, race and ethnicity, and original reason for Medicare eligibility. 95% confidence intervals constructed using standard errors clustered by county.

Source: Master Beneficiary Summary File 2010-2019 enhanced 5% sample, CMS Financial Alignment Initiative reports, American Community Survey 2019

Appendix Figure 6f: Individual event-study estimates of continuous annual Medicaid enrollment, among residents of 20% highest-poverty zip codes

Comparison group #2 (non-MMP counties in the Financial Alignment Demonstration state)

	Continuous annual Medicaid enrollment, among Medicare beneficiaries aged <65	Continuous annual Medicaid enrollment, among Medicare beneficiaries aged >=65
South Carolina		

Texas

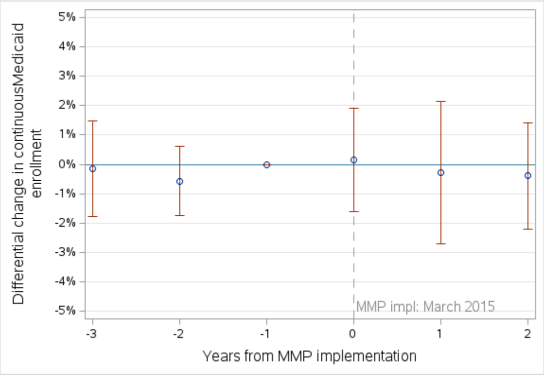

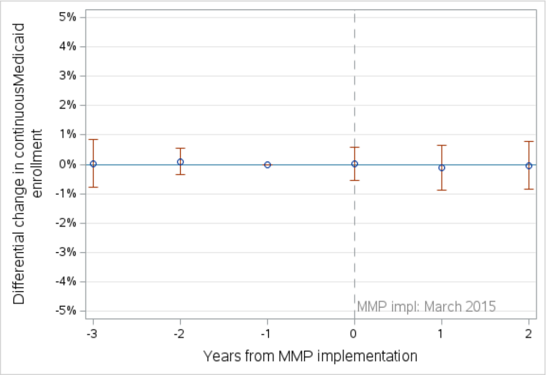

Virginia

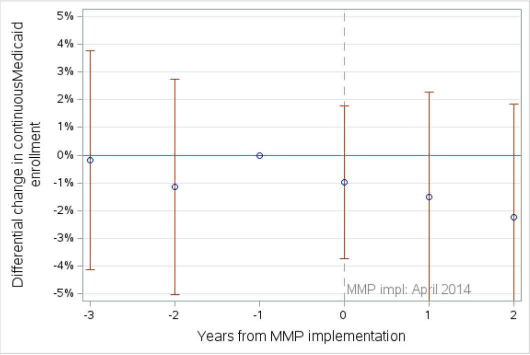

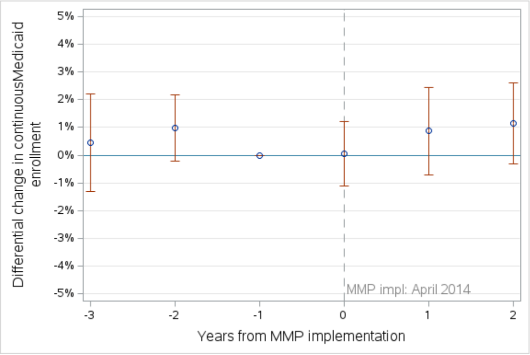

Note: Some Y-axes are differently scaled to accommodate varied differential ranges. Estimates are among Medicare beneficiaries living in the 20% of zip codes with the highest poverty rates among individuals ages 65 and older. Figures depict event-study estimates of the differential change in enrollment in continuous annual full Medicaid between the treatment group (residents of counties with MMPs in Financial Alignment Demonstration states) vs. comparison groups. The outcome is a binary person-year-level indicator for enrollment in full Medicaid in months when a beneficiary was alive and enrolled in Medicare. Comparison group #1 includes Medicare beneficiaries living in non-MMP states neighboring the Financial Alignment Demonstration state, matched on ACA Medicaid expansion status (see Table 1). Comparison group #2 includes Medicare beneficiaries living in counties of the state where MMPs were not implemented (limited to 6 states where MMPs were implemented in a subset of counties). Event-study estimates obtained from a beneficiary-year-level linear regression model predicting Medicaid enrollment as a function of treatment state indicators, event-time, and the interaction of these terms. Estimates adjusted for sex, race and ethnicity, and original reason for Medicare eligibility. 95% confidence intervals constructed using standard errors clustered by county.
Source: Master Beneficiary Summary File 2010-2019 enhanced 5% sample, CMS Financial Alignment Initiative reports, American Community Survey 2019

Appendix Figure 7: Confidence intervals based on varied extrapolation of pre-treatment violation of parallel trends for estimates of differential change in monthly full Medicaid enrollment, among residents of 20% highest-poverty zip codes

Panel A: Beneficiaries under age 65, treatment group vs. comparison group #1

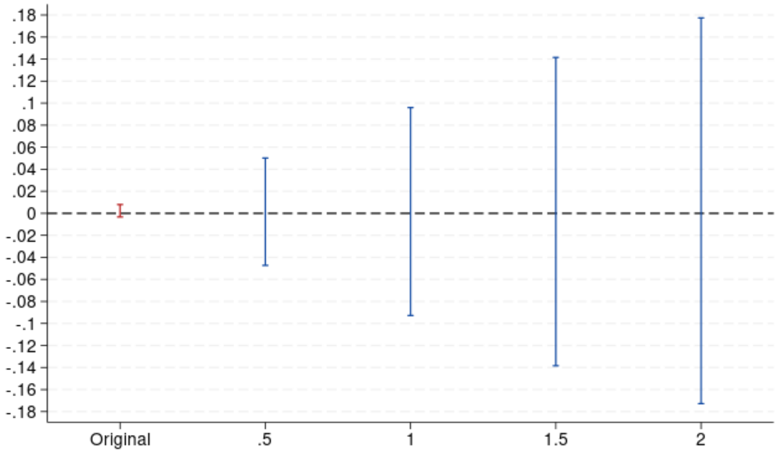

Panel B: Beneficiaries under age 65, treatment group vs. comparison group #2

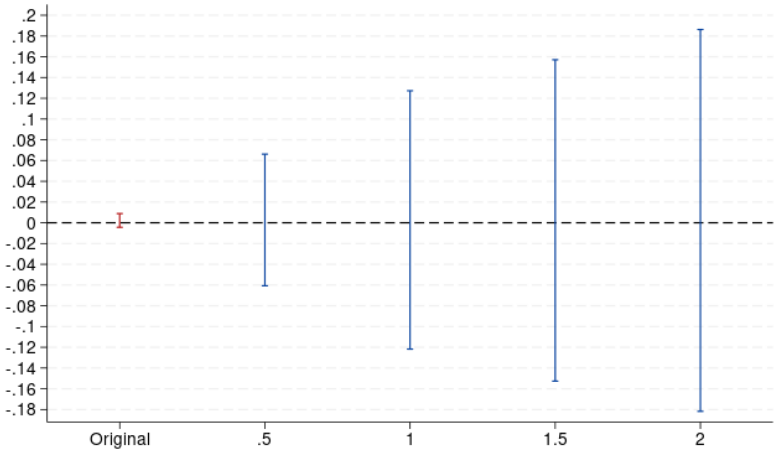

Panel C: Beneficiaries age 65 and older, treatment group vs. comparison group #1

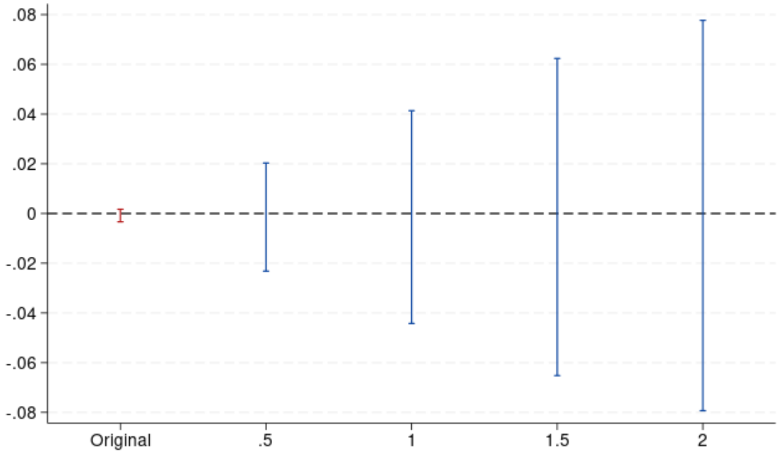

Panel D: Beneficiaries age 65 and older, treatment group vs. comparison group #2

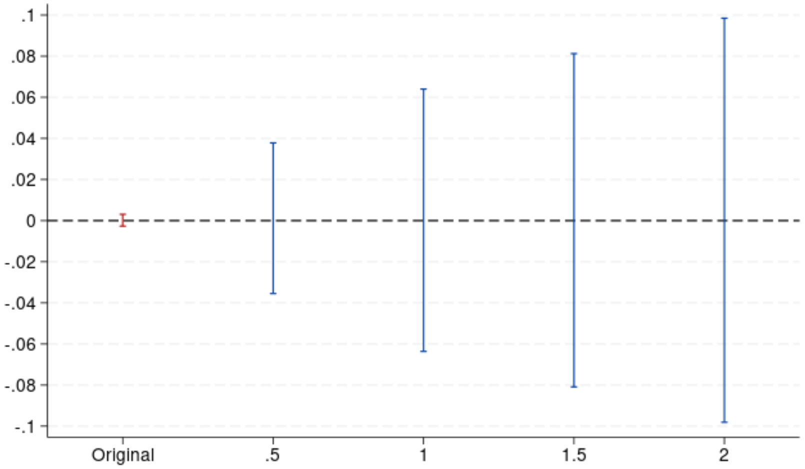

Notes: Figures depict variation based on differing extrapolations of pre-treatment violations of parallel trends in the confidence interval of the estimated differential change in enrollment in full Medicaid between the treatment group (residents of counties with MMPs in Financial Alignment Demonstration states) vs. comparison groups in the three years before vs. after MMP implementation. Analyses produced using HonestDID. Numbers on the X-axis denote bounds on the magnitude of post-treatment violations of parallel trends relative to pre-treatment violations. For example, a value of 1 imposes that the worst post-treatment violation of parallel trends between consecutive periods is no greater than the worst pre-treatment violation, while a value of 2 imposes that it the worst post-treatment violation is no more than twice as great. Note that original confidence intervals of differential change in Medicaid enrollment differ slightly in this analysis from true confidence intervals (reported in Table 3) because event-months are uniformly weighted rather than proportionally weighted in this sensitivity analysis. The outcome of the linear regression model is a binary person-month-level indicator for enrollment in full Medicaid in months when a beneficiary was alive and enrolled in Medicare. Comparison group #1 includes Medicare beneficiaries living in non-MMP states neighboring those that implemented MMPs, matched on ACA Medicaid expansion status (see Table 1). Comparison group #2 includes Medicare beneficiaries living in counties of states where MMPs were not implemented (limited to 6 states where MMPs were implemented in a subset of counties). Event-study estimates obtained from a beneficiary-month-level linear regression model predicting Medicaid enrollment as a function of treatment state indicators, event-time, and the interaction of these terms. Estimates adjusted for sex, race and ethnicity, and original reason for Medicare eligibility. 95% confidence intervals constructed using standard errors clustered by county. Panels A-B are among beneficiaries under age 65 in the study period and Panels C-D are among beneficiaries ages 65 and older in the study period.
Source: Master Beneficiary Summary File 2010-2019, American Community Survey 2019, CMS Financial Alignment Initiative reports, HonestDID Stata package

Appendix Table 4: Heterogeneity of difference-in-differences estimates of monthly Medicaid enrollment, among residents of 20% highest-poverty zip codes

Panel A. Monthly Medicaid enrollment, among beneficiaries < 65 years

	**Treatment group vs. comparison group #1** (a)				**Treatment group vs. comparison group #2** (b)			
	Differential trend pre-MMP implementation (c)		**DID estimate,** post vs. pre-MMP implementation		Differential trend pre-MMP implementation (c)		**DID estimate,** post vs. pre-MMP implementation	
	Estimate, percentage points (pp)	*95% CI*	*Estimate, pp*	*95% CI*	*Estimate, pp*	*95% CI*	*Estimate, pp*	*95% CI*
Pooled DID estimate (d)	0.36	0.13, 0.59	0.85	0.18, 1.52	0.05	-0.25, 0.36	0.50	-0.11, 1.11
Subgroup estimates:								
Beneficiaries with 1+ hospitalization(s) in prior 12 months (e)	0.56	0.07, 1.06	-0.36	-1.34, 0.63	0.18	-0.61, 0.97	-0.27	-1.48, 0.93
Beneficiaries with 0 hospitalizations in prior 12 months	0.41	0.14, 0.69	1.19	0.52, 1.87	0.09	-0.21, 0.40	0.77	0.12, 1.42

Panel B. Monthly Medicaid enrollment, among beneficiaries ≥ 65 years

	**Treatment group vs. comparison group #1** (a)				**Treatment group vs. comparison group #2** (b)			
	Differential trend pre-MMP implementation (c)		**DID estimate,** post vs. pre-MMP implementation		Differential trend pre-MMP implementation (c)		**DID estimate,** post vs. pre-MMP implementation	
	Estimate, pp	*95% CI*	*Estimate, pp*	*95% CI*	*Estimate, pp*	*95% CI*	*Estimate, pp*	*95% CI*
Pooled DID estimate (d)	-0.08	-0.25, -0.10	-0.16	-0.58, 0.25	-0.02	-0.18, 0.13	-0.05	-0.43, 0.33
Subgroup estimates:								
Beneficiaries with 1+ hospitalization(s) in prior 12 months (e)	-0.23	-0.54, 0.09	-0.43	-1.17, 0.31	0.18	-0.17, 0.54	0.12	-0.66, 0.90
Beneficiaries with 0 hospitalizations in prior 12 months	-0.04	-0.22, 0.15	-0.09	-0.49, 0.31	-0.06	-0.22, 0.11	-0.04	-0.39, 0.31
Beneficiaries aged 65-69	-0.11	-0.41, 0.19	-0.03	-0.59, 0.53	-0.16	-0.41, 0.08	-0.16	-0.77, 0.46
Beneficiaries aged 70-74	-0.15	-0.40, 0.10	-0.32	-0.85, 0.22	0.01	-0.29, 0.31	0.10	-0.52, 0.72

Beneficiaries aged 75-79	0.03	-0.24, 0.29	-0.54	-1.10, 0.02	-0.02	-0.31, 0.27	-0.48	-1.09, 0.12
Beneficiaries aged 80+	-0.01	-0.23, 0.21	0.24	-0.26, 0.74	0.08	-0.13, 0.29	0.27	-0.17, 0.71

Notes: Difference-in-differences (DID) estimates reflect differential change in enrollment in full Medicaid between the treatment groups (residents of counties with MMPs in Financial Alignment Demonstration States) vs. comparison groups for these states (see methods for details). The outcome is a binary person-month-level indicator for enrollment in full Medicaid in months when a beneficiary was alive and enrolled in Medicare. Estimates are in percentage points (pp). Estimates were obtained from a beneficiary-month-level linear regression model predicting Medicaid enrollment as a function of treatment state indicators, event-time, and the interaction of these terms. Estimates are adjusted for sex, race and ethnicity, and original reason for Medicare eligibility. 95% confidence intervals constructed using standard errors clustered by county. Shaded cells denote where analyses were not conducted because the MMP was not implemented in the age group shown (e.g., Massachusetts' MMP did not cover beneficiaries aged $\geq$65 years) or because a comparison group was not available.

(a) Comparison group #1 includes Medicare beneficiaries living in non-MMP states neighboring states that implemented MMPs, matched on ACA Medicaid expansion status (see methods for details).

(b) Comparison group #2 includes Medicare beneficiaries living in non-MMP counties in Financial Alignment Demonstration states. This analysis excludes Massachusetts, South Carolina, and Rhode Island, whose MMP programs are statewide or nearly statewide.

(c) Differential pre-MMP implementation trend reported as the annual average differential change in enrollment in full Medicaid between treatment and comparison groups during the 3-year period prior to MMP implementation.

(d) Full population difference-in-differences estimates include all observations in the pooled model.

(e) Population-specific estimates are obtained from separate models fitted on observations fitting the specified population from treatment and comparison groups.

Sources: Master Beneficiary Summary File 2010-2019 20% sample, American Community Survey 2019, CMS Financial Alignment Initiative reports

Appendix Figure 8: Within-state treatment county level median MMP enrollment rate in the 36 months following MMP implementation

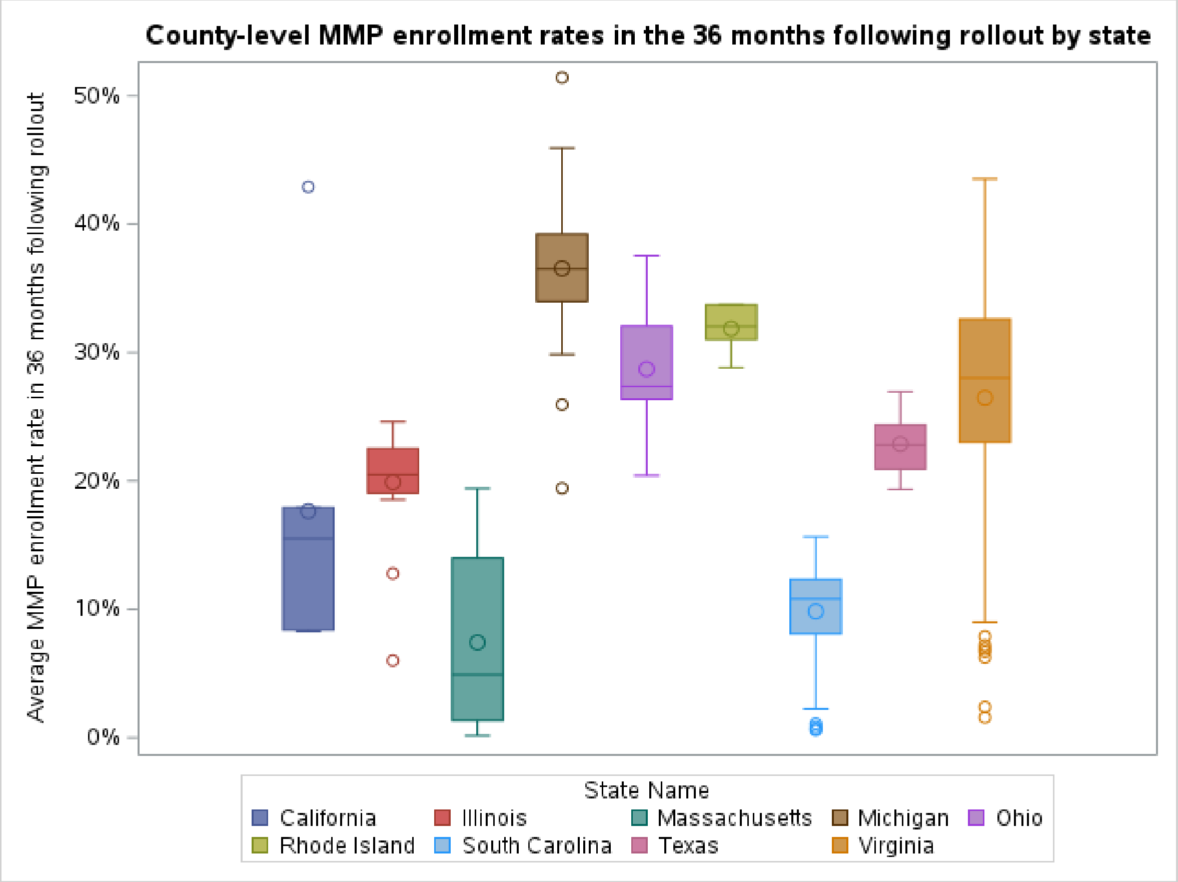

Notes: This figure reports the distribution of the average MMP enrollment rate by county aggregated across the 36 months following MMP rollout. The box represents the interquartile range; the line within the box represents the median; the circle within the box represents the mean; the whiskers represent the most extreme points within 1.5 times the interquartile range in Q1 and Q4; points outside the whisker range represent outliers. Each county contributes equal weight. Counties not offering MMPs are excluded. Eligible beneficiaries are defined as full dual residents of the county. In Massachusetts and South Carolina, eligible beneficiaries are limited to those aged <65 and >65, respectively, to account for additional eligibility requirements in those states. In Ohio, the MMP enrollment rate is aggregated across the 24 months following MMP rollout due to an eligibility rule change 27 months after MMP rollout.
Source: Master Beneficiary Summary File 20% sample 2010-2019, CMS Financial Alignment Initiative Reports

Appendix Table 5: Difference-in-differences estimates of monthly and continuous annual Medicaid enrollment limited to high MMP penetration treatment counties, among residents of 20% highest-poverty zip codes

Panel A. Monthly Medicaid enrollment

	**Treatment group vs. comparison group #1 (a)**				**Treatment group vs. comparison group #2 (b)**			
	Differential trend pre-MMP implementation (c)		**DID estimate,** post vs. pre-MMP implementation		Differential trend pre-MMP implementation (c)		**DID estimate,** post vs. pre-MMP implementation	
	Estimate, percentage points (pp)	*95% CI*	*Estimate, pp*	*95% CI*	*Estimate, pp*	*95% CI*	*Estimate, pp*	*95% CI*
Pooled DID estimate among beneficiaries aged <65	0.32	0.01, 0.63	0.46	-0.53, 1.46	-0.05	-0.39, 0.29	-0.18	-1.00, 0.65
Pooled DID estimate among beneficiaries aged ≥65	-0.16	-0.37, 0.06	-0.32	-0.97, 0.32	-0.08	-0.29, 0.12	-0.13	-0.75, 0.49

Panel B. Continuous Medicaid enrollment

	**Treatment group vs. comparison group #1 (a)**				**Treatment group vs. comparison group #2 (b)**			
	Differential trend pre-MMP implementation (c)		**DID estimate,** post vs. pre-MMP implementation		Differential trend pre-MMP implementation (c)		**DID estimate,** post vs. pre-MMP implementation	
	Estimate, pp	*95% CI*	*Estimate, pp*	*95% CI*	*Estimate, pp*	*95% CI*	*Estimate, pp*	*95% CI*
Pooled DID estimate among beneficiaries aged <65	0.85	0.35, 1.36	0.83	-0.40, 2.05	0.77	0.05, 1.49	0.08	-1.17, 1.32
Pooled DID estimate among beneficiaries aged ≥65	-0.13	-0.48, 0.21	-0.27	-1.04, 0.51	-0.01	-0.58, 0.57	-0.11	-0.86, 0.65

Notes: Difference-in-differences (DID) estimates reflect differential change in monthly and continuous annual enrollment in full Medicaid (while alive) between the treatment groups (residents of counties with MMPs in Financial Alignment Demonstration states, limited to counties with above-state-median MMP enrollment rates) vs. comparison groups for these states (see methods for details). The outcome for panel A is a binary person-month-level indicator for enrollment in full Medicaid while a beneficiary was alive and enrolled in Medicare. The outcome for panel B is a binary person-year-level indicator for enrollment in full Medicaid in all months while a beneficiary was alive and enrolled in Medicare. Estimates are in percentage points (pp). Estimates were obtained from beneficiary-month-level and beneficiary-year-level linear regression models predicting Medicaid enrollment as a function of treatment state indicators, event-time, and the interaction of these terms. Estimates are adjusted for sex, race and ethnicity, and original reason for Medicare eligibility. 95% confidence intervals constructed using standard errors clustered by county. Shaded cells denote where analyses were not conducted because the MMP was not implemented in the age group shown (e.g., Massachusetts' MMP for beneficiaries ages >65 years) or because a comparison group was not available.

(a) Comparison group #1 includes Medicare beneficiaries living in non-MMP states neighboring states that implemented MMPs, matched on ACA Medicaid expansion status (see methods for details).

(b) Comparison group #2 includes Medicare beneficiaries living in non-MMP counties in Financial Alignment Demonstration states. This analysis excludes Massachusetts, South Carolina, and Rhode Island, whose MMP programs are statewide or nearly statewide.
(c) Differential pre-MMP implementation trend reported as the annual average differential change in enrollment in full Medicaid between treatment and comparison groups during the 3-year period prior to MMP implementation.
(d) Pooled difference-in-differences estimates aggregate over the state-specific estimates using the method of stacked difference-in-differences.
Sources: Master Beneficiary Summary File 2010-2019 20% sample and enhanced 5% sample, American Community Survey 2019, CMS Financial Alignment Initiative reports
